# Enhancement of Drought Stress Tolerance in Lettuce by *Pseudomonas fluorescens* FSO7 Isolated from *Artemisia ordosica* Rhizosphere

**DOI:** 10.3390/plants15193039

**Published:** 2026-10-04

**Authors:** Ying Li, Wen Zhu, Yingao Yin, Wantong Zhao, Xiangwei He

**Affiliations:** College of Biological Sciences and Technology, Beijing Forestry University, Beijing 100083, China; yingl@bjfu.edu.cn (Y.L.);

**Keywords:** drought stress, lettuce, *Pseudomonas fluorescens*, carbon metabolism, flavonoid biosynthesis

## Abstract

Drought is a major abiotic constraint on plant productivity, and plant growth-promoting rhizobacteria (PGPR) provide a sustainable strategy for improving crop drought tolerance. The rhizosphere of the dominant Mu Us Desert plant *Artemisia ordosica* may harbor drought-adapted microorganisms, but their functions remain insufficiently characterized. In this study, drought-tolerant rhizobacteria isolated from the A. ordosica rhizosphere were screened for growth-promoting effects on lettuce (*Lactuca sativa* L.) under controlled pot conditions. The dominant strain FSO7, identified as *Pseudomonas fluorescens*, exhibited the strongest effects. Under drought stress, FSO7 inoculation significantly increased plant height, stem width, fresh weight, and dry weight by 26.62%, 111.29%, 310.19%, and 44.02%, respectively, relative to uninoculated controls. Integrated transcriptomic and untargeted metabolomic analyses showed that FSO7 inoculation was associated with marked changes in lettuce carbon metabolism under drought, involving genes and metabolites related to starch and sucrose metabolism, glycolysis, and the TCA cycle, concurrently with up-regulation of genes in the flavonoid biosynthetic pathway. These findings suggest that *P. fluorescens* FSO7 may alleviate drought-induced growth inhibition in lettuce through coordinated modulation of carbon metabolism and flavonoid-mediated antioxidant metabolism. This strain represents a promising microbial resource for developing bioinoculants for water-limited agriculture.

## 1. Introduction

Plant growth and development can be affected by a variety of biotic and abiotic stresses. Among these, drought is one of the most damaging and complex constraints on crop productivity. Water deficit induces stomatal closure, disrupts primary metabolism, and promotes oxidative stress, thereby inhibiting plant growth and causing substantial yield losses [1,2,3]. Current agricultural strategies aimed at improving plant drought tolerance mainly include breeding drought resistant varieties, plastic-film cultivation, and nanotechnology-mediated drought resistance improvement. However, these approaches can be technically complex, costly, or difficult to implement at large scale [4,5]. Therefore, green and sustainable regulatory strategies have attracted increasing attention. In particular, the construction of beneficial plant-microbe interaction systems using plant growth-promoting rhizobacteria (PGPR) has become an important strategy for enhancing plant tolerance to drought [6].

PGPR are beneficial microorganisms that colonize the rhizosphere and improve host plant performance [7,8]. Previous studies have shown that PGPR can improve plant water-use efficiency and drought tolerance by optimizing root architecture, activating antioxidant defense systems, synthesizing phytohormones, and secreting secondary metabolites [4,9,10,11,12]. *Pseudomonas fluorescens* is a representative PGPR species that can widely colonize plant ecological niches, and alleviate drought stress by strengthening the adhesion between plant roots and soil and improving soil water content [13,14]. This bacterium can also synthesize phytohormones, including auxins and cytokinins, as well as amino acids and siderophores, and can suppress soil-borne pathogens by secreting antimicrobial compounds [15,16]. Studies have confirmed that maize pretreated with *Pseudomonas* strains and lentil inoculated with *P. fluorescens* exhibit reduced drought-induced damage through the accumulation of compatible solutes [15,17]. In rice subjected to drought stress, inoculation with *P. fluorescens* upregulates the expression of the *COX1* gene involved in regulating energy and carbohydrate metabolism [16].

Different plant species often harbor distinct PGPR communities [18]. Under the selective pressure of long-term environmental stress, desert plants in arid regions have evolved specialized morphological traits and strong stress resistance [19], making them valuable natural reservoirs for screening drought-tolerant microorganisms. For example, *Bacillus* sp. WM13-24 and *Pseudomonas* sp. isolated from the rhizosphere of *Haloxylon ammodendron* in the Tengger Desert, Northwest China, enhanced drought tolerance of ryegrass by regulating phytohormones and improving antioxidant enzyme activities; WM13-24 increased root activity by 99% [19]. These findings suggest that desert-derived microorganisms may have distinctive advantages for improving plant adaptation to water deficit.

The Mu Us Desert is a typical transitional zone from semi-arid to arid in northern China, where vegetation systems have developed unique adaptive mechanisms to long-term drought stress [20]. *Artemisia ordosica*, the most prominent native plant species in the Mu Us Desert [21], may selectively recruit microbial communities that promote growth and stress resistance in nutrient-poor sandy soils [22]. In our previous study, high-throughput analysis of the *A. ordosica* rhizosphere microbiome showed that *Deinococcus* and *Streptomyces* were enriched in the rhizosphere, with *Proteobacteria* representing the dominant core group (46.43%) [23]. These microorganisms are promising candidates for developing microbial agents that improve plant stress resistance. Nevertheless, drought-tolerant microorganisms from this system remain insufficiently studied, particularly with respect to the molecular mechanisms by which they regulate plant drought responses.

A suitable host plant is required to evaluate the application potential of desert-derived PGPR. Lettuce is a leafy vegetable of the Asteraceae family [24]. With a short growth cycle and high cultivation efficiency, it is one of the most consumed leafy vegetables worldwide [25]. However, lettuce has a tissue water content exceeding 94%, a shallow primary root system, and a high transpiration rate, making it highly sensitive to drought. Even short-term water deficit can cause leaf wilting, reduced leaf area, and eventually severe yield loss [26]. These characteristics make lettuce a suitable model for studying PGPR-mediated plant drought resistance mechanisms. Current research on the interaction between *Pseudomonas fluorescens* and lettuce has predominantly focused on the biocontrol effects of this bacterium against *Escherichia coli* in lettuce, or its combination with other strains as a composite microbial agent to promote lettuce growth [27,28]. However, whether *Pseudomonas fluorescens* exerts growth-promoting effects on lettuce under drought stress remains insufficiently explored.

Based on these considerations, our team previously screened three potential strains from the roots of *A. ordosica*: *Pseudomonas fluorescens* FSO7, *Pseudomonas fluorescens* FSO11, and *Bacillus brevis* CR30. Preliminary functional analyses indicated that all three strains exhibited favorable PGPR traits and drought tolerance potential (Table S1). Although all three strains were initially considered as candidates, only the strain with the strongest growth-promoting effect under drought stress was selected for in-depth transcriptomic and metabolomic analyses.

On this basis, using lettuce as a model plant, this study employed pot experiments combined with integrated transcriptomic and metabolomic analyses to address the following two key questions: (1) whether the three-candidate growth-promoting strains derived from *A. ordosica* roots could enhance lettuce growth and drought tolerance, and which strain was the most effective; and (2) what molecular regulatory mechanisms underlie the growth promotion and drought tolerance induced by the most effective strain. Our findings will provide a basis for understanding interactions between drought-tolerant microorganisms and plants and for exploiting desert plant-associated microbial resources in sustainable agriculture.

## 2. Results

### 2.1. FSO7 Inoculation Improves Lettuce Growth Under Drought Stress

To evaluate the growth-promoting effects of different bacterial strains on lettuce under drought stress, five treatments were compared under both well-watered and drought conditions: uninoculated control (N), FSO7 (Pf), CR30, FSO11, and the combined CR30+FSO11. Two-way ANOVA revealed significant main effects of water treatment and strain treatment, as well as a significant water × strain interaction, on most growth traits (Table 1). Water treatment explained the largest proportion of total variation for whole-plant fresh weight (80.05%), shoot fresh weight, plant height, and stem width, indicating that drought was the dominant factor affecting lettuce growth (Table 1).

**Figure 1:**
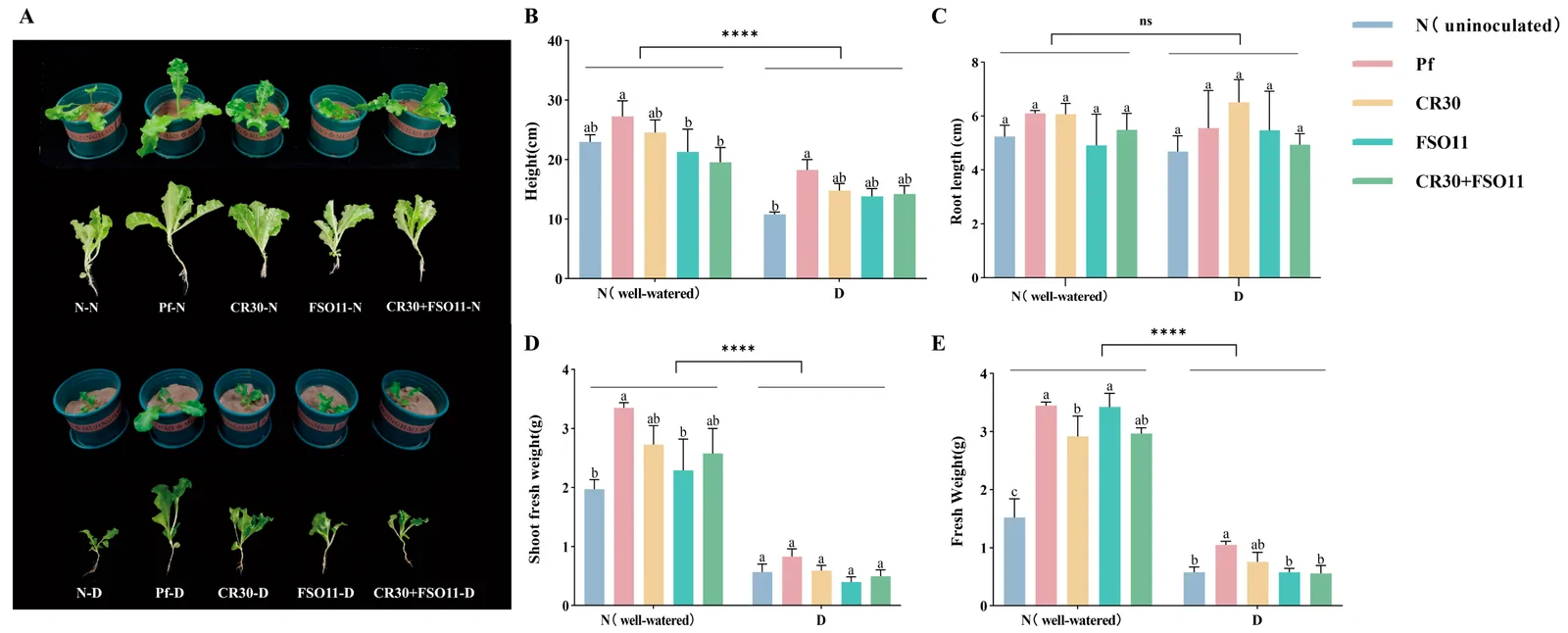
Effects of five bacterial treatments on lettuce growth under well-watered and drought conditions. (**A**) Representative phenotypes of lettuce seedlings under uninoculated control (N), FSO7 (Pf), CR30, FSO11, and CR30+FSO11 under well-watered (top) and drought (bottom) conditions. (**B**–**E**) Plant height (**B**), root length (**C**), shoot fresh weight (**D**), and whole-plant fresh weight (**E**). Data were analyzed by two-way ANOVA followed by Tukey’s multiple-comparison test. Different lowercase letters indicate significant differences among strain treatments within each water regime (*p* < 0.05). Error bars indicate SD (*n* = 3). ****, *p* < 0.0001; ns, not significant.

Morphological observations were generally consistent with the quantitative measurements. Under well-watered conditions, all five treatments produced healthy plants, and FSO7-inoculated plants (Pf-N) showed better growth than the other strain treatments (Figure 1A, top panel). Under drought stress, uninoculated plants (N-D) were stunted and slender with fewer and smaller leaves, whereas all bacterial treatments improved lettuce growth to varying degrees, with FSO7-inoculated plants (Pf-D) showing the best overall growth (Figure 1A, bottom panel).

Among the five treatments, FSO7 consistently showed the strongest growth-promoting effect under both water regimes (Figure 1B–E). Under well-watered conditions, FSO7-inoculated plants had the highest shoot fresh weight and whole-plant fresh weight among all treatments, significantly higher than the uninoculated control and CR30+FSO11 (*p* < 0.05, Tukey’s test; Figure 1D,E). Under drought stress, FSO7-inoculated plants had the highest plant height, stem width, shoot fresh weight, and whole-plant fresh weight among all bacterial treatments (Figure 1B–E). Notably, FSO7-inoculated plants had significantly higher plant height than the uninoculated drought control (*p* < 0.05; Figure 1B), and significantly higher shoot fresh weight than both the uninoculated control and CR30+FSO11 (*p* < 0.05; Figure 1D). In contrast, CR30, FSO11, and CR30+FSO11 did not differ significantly from the uninoculated control in most shoot traits under drought stress (Figure 1B–E).

Root length was also measured to assess the effect of bacterial inoculation on root architecture. However, two-way ANOVA showed no significant main effects of water treatment (F (1,20) = 0.1742, *p* > 0.05) or strain treatment (F (4,20) = 2.437, *p* > 0.05), nor a significant water × strain interaction (F (4,20) = 0.6762, *p* > 0.05) on root length (Table 1; Figure 1C). These results suggest that root length remained relatively stable across treatments under our experimental conditions, and that the growth-promoting effect of FSO7 was may be mainly reflected in shoot traits.

To further quantify the effect of FSO7 inoculation relative to the uninoculated control, a separate two-way ANOVA was performed comparing N and Pf under both water regimes (Table S2; Figure S1A–E). The results showed significant main effects of water treatment on plant height (F (1,20) = 19.67, *p* < 0.001), stem width (F (1,20) = 15.26, *p* < 0.001), and fresh weight (F (1,20) = 21.42, *p* < 0.001), and a significant water × strain interaction on stem width (F (1,20) = 8.804, *p* < 0.01), dry weight (F (1,20) = 12.54, *p* < 0.01), and fresh weight (F (1,20) = 12.95, *p* < 0.01). Under drought stress, FSO7 inoculation significantly increased plant height (*p* < 0.01; Figure S1A), stem width (*p* < 0.01; Figure S1C), and fresh weight (*p* < 0.01; Figure S1E) compared with the uninoculated control, while no significant difference was detected under well-watered conditions for most traits. These results suggest that the growth-promoting effect of FSO7 was more pronounced under drought stress than under well-watered conditions, and that this effect was mainly reflected in shoot traits.

Compared with the well-watered uninoculated control (N-N), plant height, stem width, fresh weight, and dry weight in drought-stressed lettuce (N-D) decreased by 25.28%, 56.82%, 84.64%, and 42.23%, respectively. After FSO7 inoculation under drought stress, plant height, stem width, fresh weight, and dry weight increased by 26.62%, 111.29%, 310.19%, and 44.02%, respectively, compared with the uninoculated drought-stressed control (Figure S1A–E). Based on these growth traits and the two-way ANOVA results, FSO7 was selected as the most promising candidate among the tested strains for subsequent integrated transcriptomic and metabolomic analyses.

### 2.2. Transcriptome Analysis of Lettuce Inoculated with FSO7 Under Drought Stress

#### 2.2.1. Transcriptome Sequencing Results

Lettuce leaves from the N-N, N-D, Pf-N, and Pf-D treatments were used for transcriptomic analysis. Clean reads from each sample were aligned to the lettuce reference genome, with mapping rates ranging from 97.40% to 97.99%. These results indicated that the sequencing data were of high quality and suitable for downstream bioinformatic analysis (Table S3). Pearson correlation analysis showed high consistency among biological replicates within each treatment and clear differences among treatment groups (Figure S2A). Based on these sequencing data, differentially expressed gene (DEG) analysis was performed to evaluate the regulatory effect of *Pseudomonas* FSO7 on the lettuce leaf transcriptome under drought stress.

#### 2.2.2. Identification of Differentially Expressed Genes (DEGs)

To characterize transcriptional responses under different treatments, four pairwise comparisons were analyzed: N-N vs. N-D, N-N vs. Pf-N, Pf-N vs. Pf-D, and Pf-D vs. N-D. DEGs were screened using thresholds of |log_2_(fold change)| ≥ 1 and *p* < 0.05. Venn diagram analysis illustrated the shared and specific DEGs among the four comparisons (Figure S2B). In the N-N vs. N-D comparison, 8211 DEGs were identified, including 4058 upregulated and 4153 downregulated genes. In the N-N vs. Pf-N comparison, 850 DEGs were identified, including 602 upregulated and 248 downregulated genes. In the Pf-N vs. Pf-D comparison, 6713 DEGs were identified, including 2972 upregulated and 3731 downregulated genes. In the Pf-D vs. N-D comparison, 1554 DEGs were identified, including 635 upregulated and 919 downregulated genes (Figure S2C).

#### 2.2.3. GO Annotation and KEGG Pathway Enrichment of DEGs

**Figure 2:**
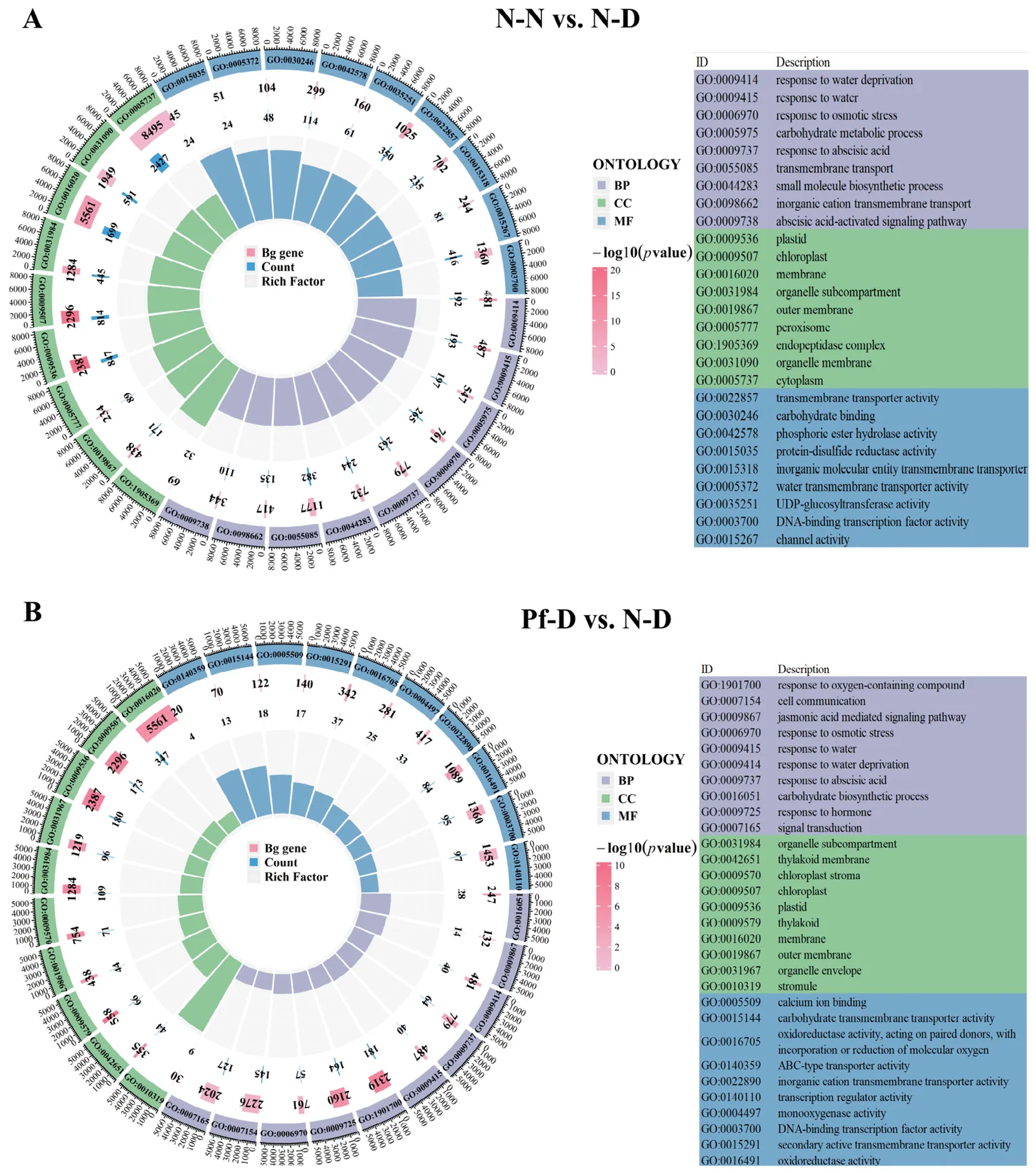
GO enrichment analysis of DEGs in representative comparisons. (**A**) N-N vs. N-D. (**B**) Pf-D vs. N-D. BP, biological process; CC, cellular component; MF, molecular function.

GO enrichment analysis was performed to functionally characterize DEGs under different drought-stress conditions. Under drought treatment, DEGs in the N-N vs. N-D and Pf-N vs. Pf-D comparisons were mainly enriched in biological processes related to transmembrane transport, response to abscisic acid, response to water, and response to water deprivation. Enriched molecular functions included DNA-binding transcription factor activity and UDP-glycosyltransferase activity (Figure 2A and Figure S3A). Under well-watered treatment, FSO7 influenced genes associated with abiotic stress responses, responses to oxygen-containing compounds, transcriptional regulation, monooxygenase activity, and the CCR4-NOT complex (Figure S3B). Under drought stress, FSO7 enhanced the expression of genes associated with responses to oxygen-containing compounds and phytohormones, and enriched jasmonic acid (JA)-mediated signaling and ABC-type transporter activity (Figure 2B). Collectively, these GO enrichment results suggest the possible involvement of antioxidant-related responses, hormone signaling, and substance transport in the observed changes associated with FSO7 inoculation under drought stress.

KEGG pathway enrichment analysis was performed to further characterize the transcriptional changes associated with FSO7 inoculation in lettuce under drought stress. In the drought comparisons with or without FSO7 inoculation (N-N vs. N-D and Pf-N vs. Pf-D), DEGs were significantly enriched in biosynthesis of secondary metabolites, plant hormone signal transduction, MAPK signaling, carbon metabolism, arginine and proline metabolism, and glutathione metabolism (Figure S4A,B). These pathways are associated with water-deficit responses through hormone signaling, particularly ABA-related signaling, osmolyte accumulation, antioxidant defense, a metabolic reallocation. Under well-watered supply, FSO7 inoculation was associated with enrichment of genes related to photosynthesis-antenna proteins and basal immunity (Figure S4C). In the Pf-D vs. N-D groups, the application of FSO7 affected the signaling pathway transduction of hormones, the production of antioxidants such as flavonoids and phenylpropanoids in lettuce, and photosynthetic carbon fixation in lettuce (Figure S4D).

#### 2.2.4. qRT-PCR Validation of Transcriptome Data

To validate the RNA-seq results, nine DEGs were selected for qRT-PCR analysis. These genes were chosen to span different pairwise comparisons, both up- and down-regulated directions, and a broad range of fold-change magnitudes. The expression patterns of genes encoding invertase (INV*DC4), thiamine thiazole synthase (*THI1*), stay-green protein (*SGR1*), serine/threonine-protein kinase SAPK3 (*SAPK3*), protein phosphatase 2C (*PP2CA*), pyrimidine 1 (*PYD1*), glutathione peroxidase 2 (*GPXHA-2*), ribulose-1,5-bisphosphate carboxylase small subunit (*RBCS*), and the uncharacterized gene *LOC111897065* were consistent with the RNA-seq data (Figure S5A). All nine genes showed the same direction of change between the two platforms, and the correlation was highly significant (R^2^ = 0.9792, Figure S5B). These results confirmed the reliability of the transcriptomic analysis.

### 2.3. Metabolome Analysis of Lettuce Inoculated with FSO7 Under Drought Stress

**Figure 3:**
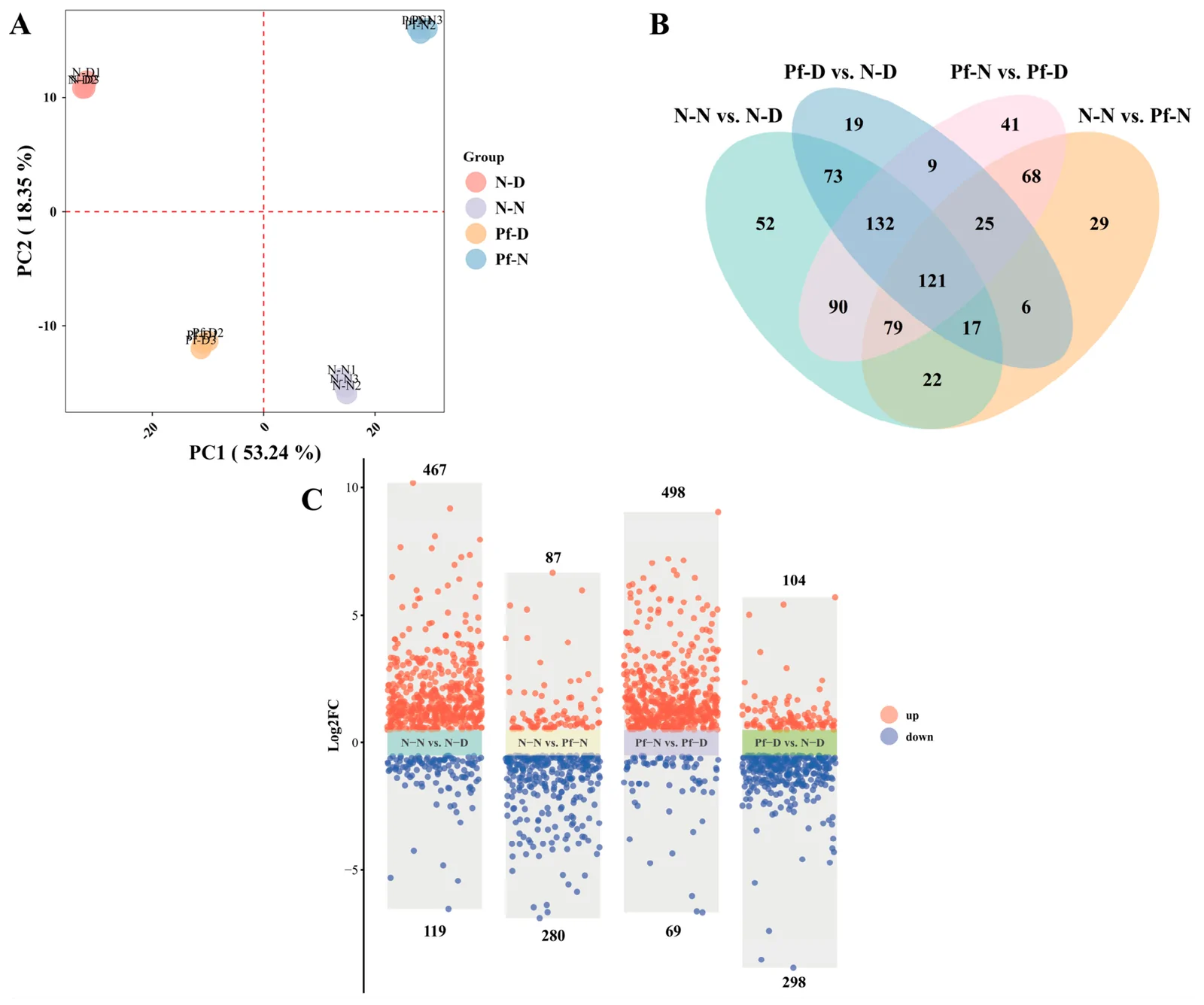
Metabolomic profiles of lettuce under FSO7 inoculation and drought stress. (**A**) Principal component analysis (PCA) of metabolomic profiles from different treatment groups. (**B**) Venn diagram showing the overlap of differentially accumulated metabolites (DAMs) among pairwise comparisons. (**C**) DAMs in the N-N vs. N-D, N-N vs. Pf-N, Pf-N vs. Pf-D, and Pf-D vs. N-D comparisons. Red indicates upregulated genes, and blue indicates downregulated genes. Numbers above and below each comparison indicate upregulated and downregulated metabolites, respectively.

To determine the effects of drought stress and FSO7 inoculation on lettuce metabolites, LC-MS/MS-based untargeted metabolomics was used to profile lettuce leaf metabolites. Principal component analysis (PCA) showed clear separation among treatment groups and tight clustering of biological replicates. Two principal components (PC1 and PC2) explained 53.24% and 18.35% of the total variation, respectively (Figure 3A). A total of 1109 metabolites were annotated. Differentially accumulated metabolites (DAMs) were screened using thresholds of |log_2_(fold change)| > 0.5, variable importance in projection (VIP) > 1, and *p* < 0.05. Venn diagram analysis showed the shared and specific DAMs among the four comparisons (Figure 3B). In the N-N vs. N-D comparison, 586 DAMs were detected, including 467 increased and 119 decreased metabolites. In the N-N vs. Pf-N comparison, 367 DAMs were detected, including 87 increased and 280 decreased metabolites. In the Pf-N vs. Pf-D comparison, 567 DAMs were detected, including 498 increased and 69 decreased metabolites. In the Pf-D vs. N-D comparison, 402 DAMs were detected, including 104 increased and 298 decreased metabolites (Figure 3C).

**Figure 4:**
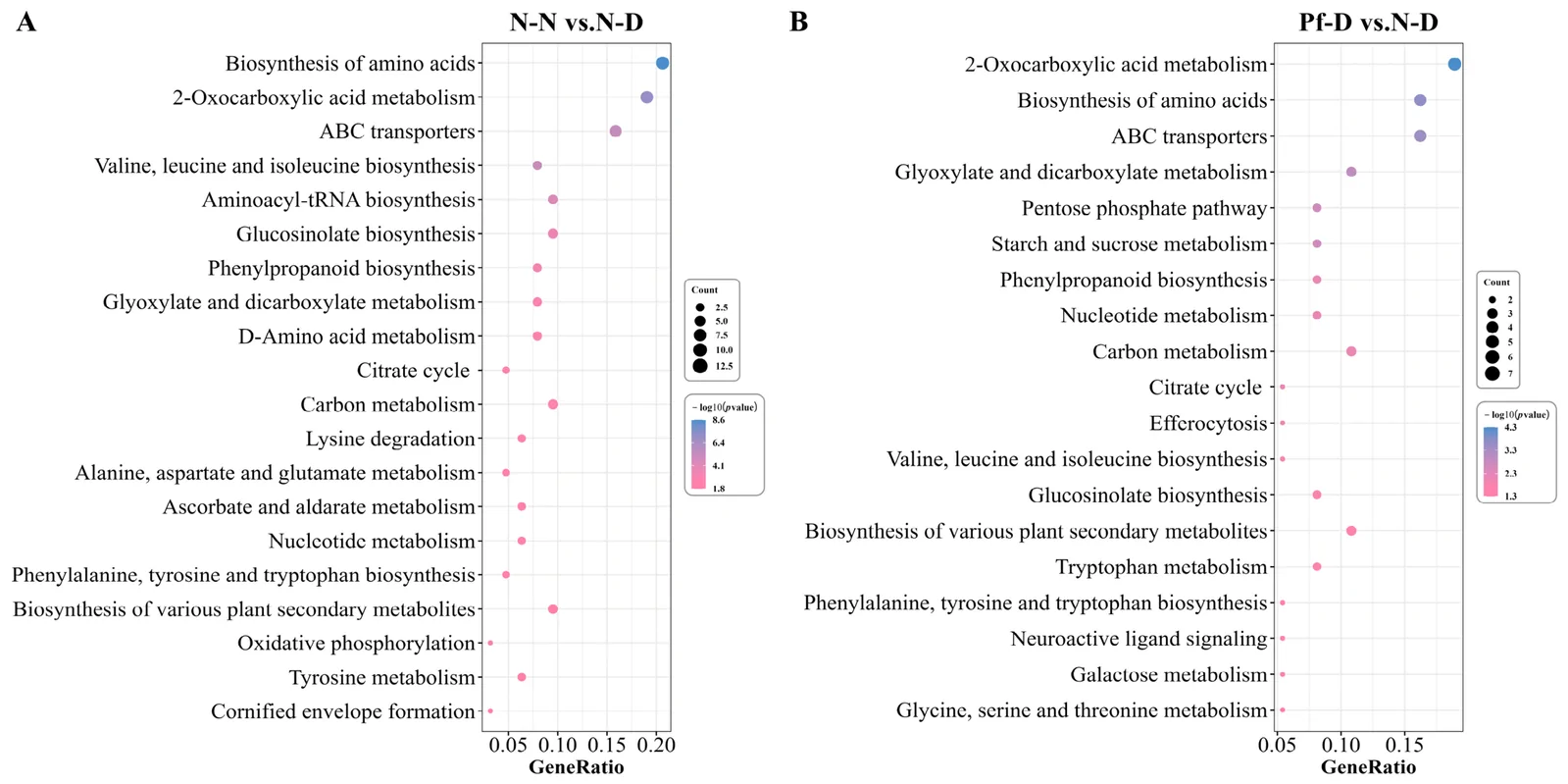
KEGG pathway enrichment of DAMs in the four pairwise comparisons. (**A**) N-N vs. N-D. (**B**) Pf-D vs. N-D. Bubble size indicates the number of DAMs enriched in each pathway, and color indicates enrichment significance.

KEGG pathway enrichment analysis was performed to identify metabolic pathways affected by drought stress and FSO7 inoculation. In the N-N vs. N-D comparison, DAMs were significantly enriched in biosynthesis of amino acids, 2-oxocarboxylic acid metabolism, ABC transporters, valine, leucine, and isoleucine biosynthesis, and aminoacyl-tRNA biosynthesis (Figure 4A). In the N-N vs. Pf-N comparison, enriched pathways included biosynthesis of amino acids, 2-oxocarboxylic acid metabolism, glyoxylate and dicarboxylate metabolism, nucleotide metabolism, and the citrate cycle (Figure S6A). In the Pf-D vs. N-D comparison, DAMs were enriched in 2-oxocarboxylic acid metabolism, biosynthesis of amino acids, ABC transporters, the pentose phosphate pathway, starch and sucrose metabolism, and phenylpropanoid biosynthesis (Figure 4B). In the Pf-N vs. Pf-D comparison, DAMs were significantly enriched in biosynthesis of amino acids, ABC transporters, nucleotide metabolism, 2-oxocarboxylic acid metabolism, and glyoxylate and dicarboxylate metabolism (Figure S6B).

### 2.4. Integrated Transcriptome and Metabolome Analysis of the Pf-D vs. N-D Comparison

To preliminarily explore the potential molecular basis underlying the improved drought performance of lettuce associated with FSO7 inoculation, transcriptomic and metabolomic data from the Pf-D and N-D groups were integrated. Nine-quadrant plot analysis showed a correlation between transcriptome and metabolome data (Figure S7A). KEGG pathway enrichment analysis of all DEGs and DAMs identified 21 co-enriched pathways, which were selected for visualization (Figure S7B). These pathways mainly included starch and sucrose metabolism, phenylpropanoid biosynthesis, flavonoid biosynthesis, and carbon metabolism. We therefore focused on two pathways enriched by both DEGs and DAMs: starch and sucrose metabolism and flavonoid biosynthesis.

**Figure 5:**
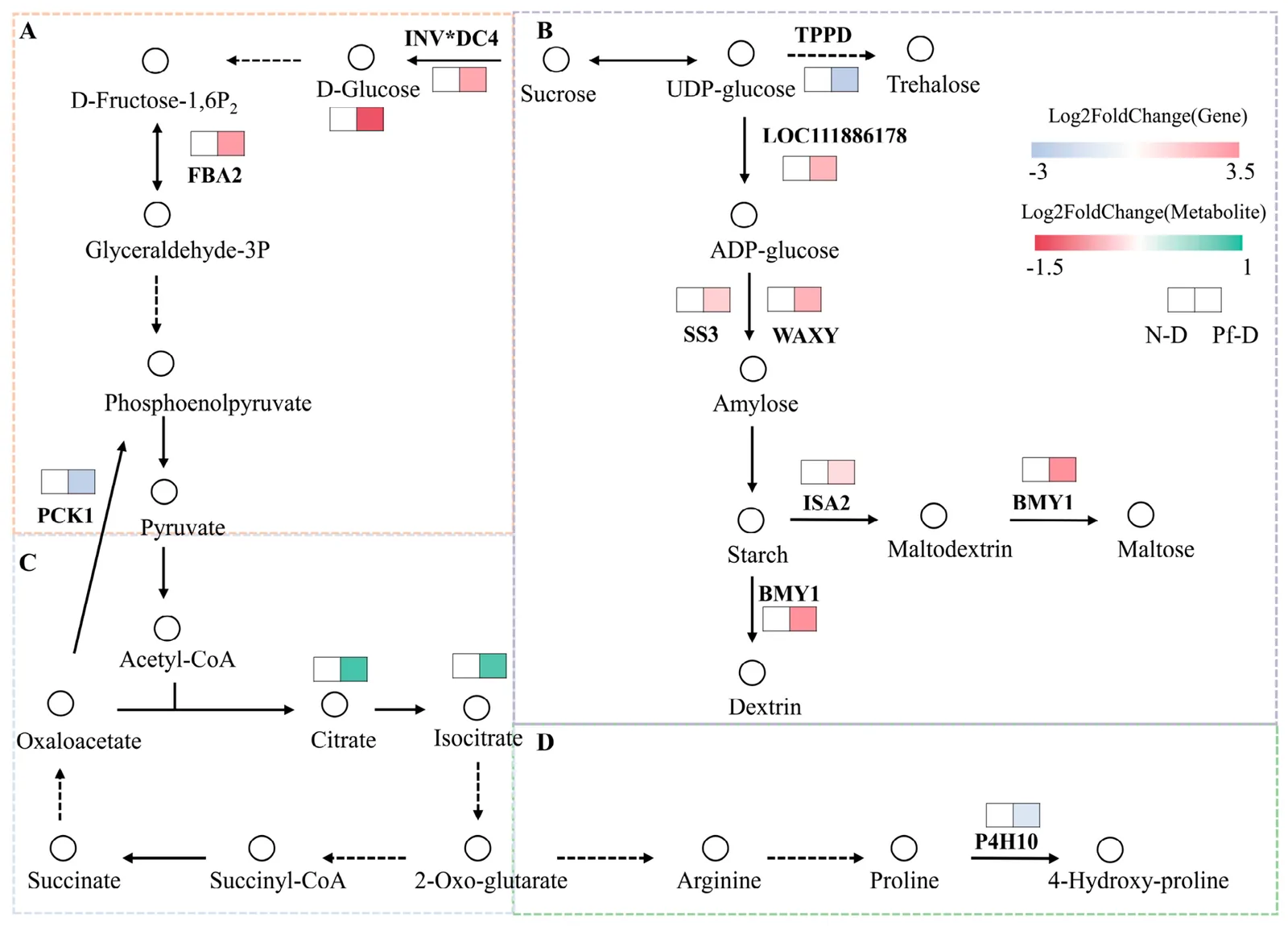
Integrated analysis of central carbon and proline-related metabolism in lettuce under FSO7 inoculation and drought stress. (**A**) Glycolysis. (**B**) Starch and sucrose metabolism. (**C**) Tricarboxylic acid (TCA) cycle. (**D**) Proline-related metabolism. Circles represent pathway nodes. Enzyme names above arrows indicate reactions associated with differentially expressed genes. Each rectangle is divided into two halves corresponding to N-D and Pf-D treatments; the color scales represent gene log_2_FC values from −3 to 3.5 and metabolite log_2_FC values from −1.5 to 1, and apply only to the data shown in this figure. Solid arrows indicate direct metabolic reactions, and dashed arrows indicate multi-step reactions.

In the starch and sucrose metabolism pathway (Figure 5B and Table S3), expression of the gene encoding trehalose 6-phosphophosphatase (*TPPD*), a key enzyme in sucrose metabolism to produce trehalose, was significantly downregulated. This result suggests that trehalose synthesis may not be the primary osmoprotective route induced by FSO7 under drought stress. Genes associated with starch synthesis, including *SS3* and *WAXY*, were upregulated. Genes encoding β-amylase 1 (*BMY1*) and invertase (INV*DC4) were also significantly upregulated; however, the abundance of D-glucose, a key product of starch and sucrose hydrolysis, was significantly reduced. Further analysis of central carbon metabolism showed that fructose-1,6-bisphosphate aldolase (*FBA2*) was upregulated in the glycolytic pathway, which may reflect enhanced conversion of glucose-derived metabolites toward pyruvate (Figure 5A and Table S3). In the tricarboxylic acid (TCA) cycle, citrate and isocitrate levels were significantly increased, whereas *PCK1*, which encodes a key gluconeogenic enzyme catalyzing the conversion of oxaloacetate to phosphoenolpyruvate, was significantly downregulated (Figure 5C and Table S3). In addition, the TCA cycle intermediate 2- oxoglutarate can contribute to proline biosynthesis through transamination reactions, whereas *P4H10*, which encodes proline 4-hydroxylase, was significantly downregulated (Figure 5D and Table S3). These combined transcriptomic and metabolomic changes suggest a coordinated involvement of starch and sucrose turnover, glycolysis, the TCA cycle, and proline-related metabolism in energy supply and osmotic adjustment processes in lettuce following FSO7 inoculation under drought stress.

**Figure 6:**
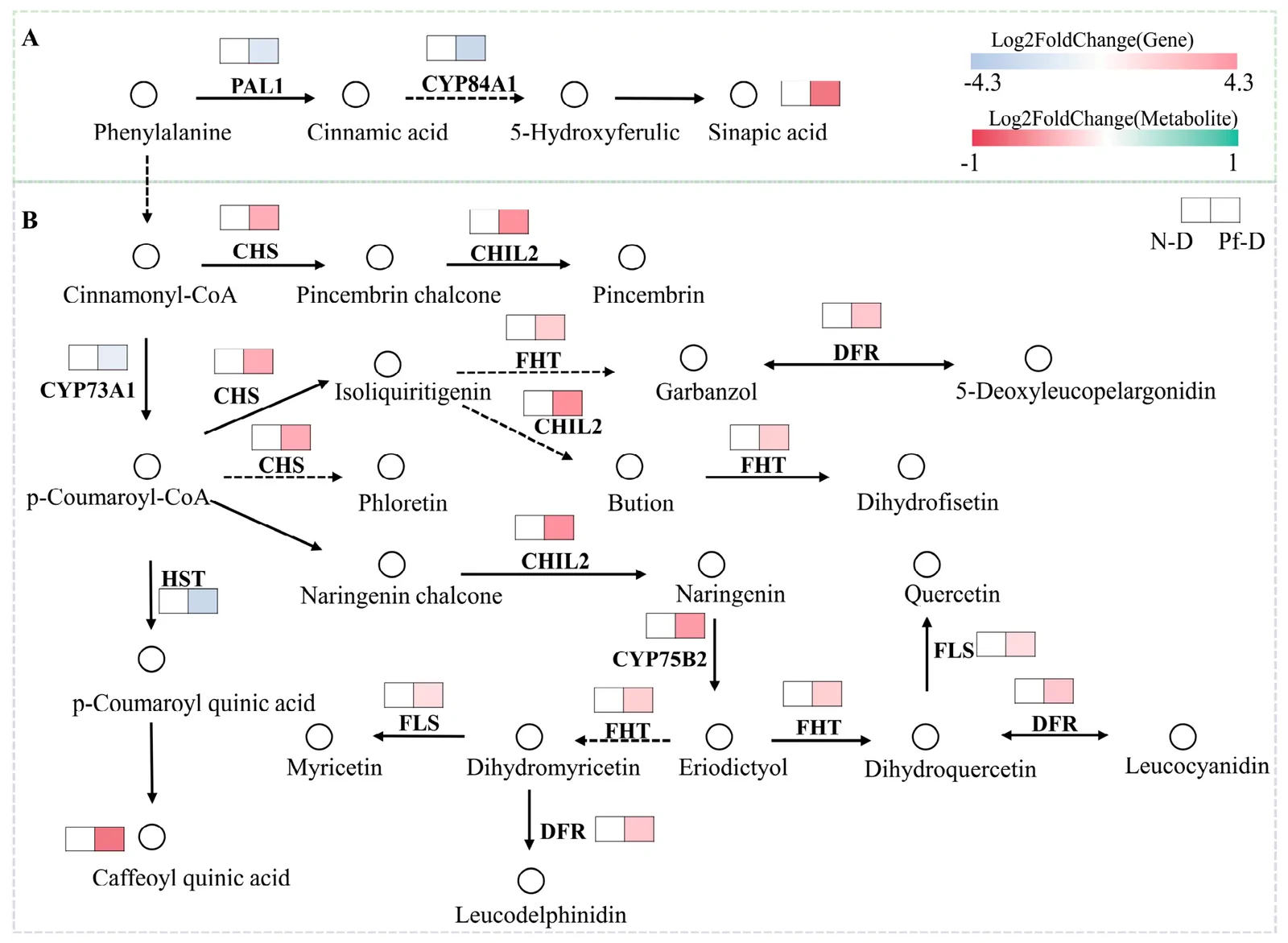
Integrated analysis of phenylpropanoid and flavonoid biosynthesis in lettuce under FSO7 inoculation and drought stress. (**A**) Phenylpropanoid biosynthesis. (**B**) Flavonoid biosynthesis. Circles represent pathway nodes, i.e., intermediates in the phenylpropanoid and flavonoid biosynthetic routes. Enzyme names above arrows indicate reactions associated with differentially expressed genes. Each rectangle is divided into two halves corresponding to N-D and Pf-D treatments; the color scales represent gene log_2_FC values from −4.3 to 4.3 and metabolite log_2_FC values from −1 to 1, and apply only to the data shown in this figure. Solid arrows indicate direct metabolic reactions, and dashed arrows indicate multi-step reactions.

In the Pf-D group, transcript levels of genes in certain branches of the phenylpropanoid pathway showed downregulation (Figure 6A and Table S4), whereas genes in the flavonoid biosynthetic pathway showed upregulation (Figure 6B and Table S4). Branching pathways downstream of phenylalanine associated with lignin synthesis were downregulated at the transcriptional level. Genes encoding phenylalanine ammonia-lyase (*PAL1*) and ferulate 5-hydroxylase (*CYP84A1*) were significantly downregulated, accompanied by a significant reduction in the downstream product sinapic acid. Genes encoding cinnamate 4-hydroxylase (*CYP73A1*) and hydroxycinnamoyl-CoA transferase (*HST*) were also downregulated, accompanied by decreased accumulation of caffeoylquinic acid. In contrast, the flavonoid biosynthetic branch showed upregulated gene expression. Flavonoids are derived from cinnamoyl-CoA and p-coumaroyl-CoA, which are core intermediates of phenylalanine metabolism. *CHIL2*, which encodes an enzyme involved in the formation of flavonoid precursors such as pinocembrin and butin, was significantly upregulated. Further analysis showed that several genes involved in flavonoid biosynthesis, including *CHS*, *CHIL2*, *FHT*, *DFR*, *FLS*, and *CYP75B2*, were significantly upregulated. These combined transcriptomic and metabolomic changes suggest that transcriptional activation of the flavonoid biosynthetic pathway may contribute to improved drought performance in lettuce following FSO7 inoculation, although the accumulation of specific antioxidant flavonoids was not confirmed in the present untargeted metabolomics analysis.

## 3. Discussion

### 3.1. Potential Links Between Strain-Level Plant Growth-Promoting Traits and the Superior Performance of FSO7

Although all three tested strains exhibited plant growth-promoting traits and drought tolerance potential in the preliminary screening (Table S1), which supported their selection as candidates, only FSO7 consistently showed the strongest growth-promoting effect on lettuce under drought stress. This was evident from the pot experiment, where FSO7-inoculated plants had significantly higher plant height, stem width, and shoot fresh weight than the uninoculated drought control, whereas FSO11 and CR30 did not differ significantly from the control in most shoot traits (Figure 1B–E). This disparity is particularly notable between the two *Pseudomonas fluorescens* strains (FSO7 and FSO11). Based on the screening data, two strain-level differences may help explain this. FSO7 produced a more moderate level of IAA (7.38 mg/L) compared to FSO11 (21.82 mg/L) and CR30 (28.05 mg/L). The effects of microbial IAA on plant growth are known to be concentration-dependent, with an optimal range beyond which growth may be inhibited [29,30]. The moderate IAA level in FSO7 may therefore provide a more balanced hormonal stimulus, potentially favoring shoot growth under drought. Additionally, FSO7 exhibited the highest siderophore production (D/d = 2.97), which may be particularly advantageous in the Mu Us Desert sandy soil where available iron is often limited [31], potentially improving iron acquisition and rhizosphere competitiveness under drought. Siderophores produced by PGPR can chelate insoluble iron in the soil and convert it into plant-available forms, thereby improving plant iron nutrition [32,33]. Based on its strongest growth-promoting effect under drought stress, FSO7 was selected for in-depth transcriptomic and metabolomic analyses. Nevertheless, further studies are needed to confirm the specific contributions of these traits under field conditions.

### 3.2. FSO7 Inoculation Is Associated with Changes in Carbohydrate, Energy, and Osmolyte-Related Metabolism in Drought-Stressed Lettuce

When plants are exposed to drought stress, their carbohydrate metabolism undergoes adaptive remodeling [34]. Glycolysis and the TCA cycle are central pathways for carbohydrate metabolism and energy supply in plants [8]. As the main storage forms of carbohydrates, starch and sucrose provide energy, maintain cell swelling to sustain plant biological processes and facilitate water absorption [35]. During drought stress, starch can be rapidly mobilized into glucose to provide immediate energy for key cellular physiological activities [36]. Sucrose can be hydrolyzed by invertase (INV) family enzymes and contribute to hexose accumulation, thereby providing sugar required for osmotic protection in plants under water deficit environment [34,37].

In this study, several genes involved in starch and sucrose metabolism were significantly upregulated in lettuce inoculated with FSO7; however, the accumulation of the end product D-glucose did not increase. One possible interpretation of this pattern is that glucose derived from carbohydrate turnover may have been channeled into downstream metabolic pathways. This interpretation is supported by the significant upregulation of the glycolytic gene *FBA2*. Furthermore, in the TCA cycle, citrate and isocitrate levels were increased, while *PCK1*, which encodes a key gluconeogenic enzyme, was downregulated. These changes may reflect a shift from carbohydrate storage and gluconeogenic recycling toward energy-generating metabolism in FSO7-inoculated plants under drought. Similar metabolic redirection has been observed in PGPR-treated plants, in which microbial inoculation altered pyruvate metabolism pathways [38].

The TCA cycle is also linked to amino acid metabolism. In particular, α-ketoglutarate is a key branch point metabolite that participates in the TCA cycle and provides carbon skeletons for amino acid synthesis [39]. Under drought stress, proline accumulation is a well-known osmoprotective response that stabilizes proteins and membranes and contributes to cellular redox balance [40]. In the present study, the gene encoding proline 4-hydroxylase was downregulated, which may suggest reduced conversion of proline to 4-hydroxyproline and possibly enhanced retention of proline-related osmoprotective capacity. Previous studies have reported that mung bean plants inoculated with *Pseudomonas fluorescens* accumulate higher proline levels under drought stress compared with non-inoculated controls [41]. On this basis, we hypothesize that FSO7 inoculation might contribute to drought tolerance in lettuce partly through modifying carbon distribution toward compatible solute-related pathways.

Trehalose is another important osmoprotectant involved in plant stress tolerance and plant-microbe symbiosis [42,43]. In our data, however, the gene encoding trehalose-6-phosphate phosphatase was significantly downregulated in the drought treatment with FSO7 inoculation. This observation suggests that, under the experimental conditions tested here, trehalose accumulation may not be the primary osmoprotective mechanism induced by FSO7, and that other osmoregulatory substances might play a more prominent role.

### 3.3. FSO7 Inoculation Is Associated with Altered Phenylpropanoid and Flavonoid Metabolism in Drought-Stressed Lettuce

Drought stress induces reactive oxygen species (ROS) accumulation in plant cells. ROS function as stress signaling molecules and can activate plant defense pathways; however, excessive ROS accumulation causes peroxidation of biological macromolecules, disrupts membrane integrity, interferes with cellular energy metabolism and biosynthesis, and may ultimately lead to cell death [44,45]. Flavonoids are important plant antioxidants that accumulate under drought stress and enhance plant antioxidant capacity by scavenging excessive ROS and alleviating oxidative damage [46]. In *Arabidopsis thaliana*, flavonoids have been identified as stress metabolites that mediate plant-microbe interactions during dehydration responses [47].

Flavonoid biosynthesis originates from the phenylpropanoid biosynthesis [48]. Drought stress is often accompanied by enhanced phenylpropanoid metabolism and lignin accumulation. For example, inoculation of *Pseudomonas aeruginosa* SAESo11 significantly upregulated the phenylpropanoid pathway in tomato under drought stress [5], and *Bacillus velezensis* D103 enhanced maize drought tolerance by activating this pathway and promoting lignin deposition [49]. However, in this study, genes encoding key enzymes involved in lignin synthesis within the phenylpropanoid pathway were significantly downregulated. The intermediate metabolites caffeoylquinic acid and sinapic acid were also reduced. Caffeoylquinic acid exhibits antioxidant activity and can improve drought tolerance in wheat [50], whereas sinapic acid is a core intermediate in lignin biosynthesis [51,52].

Concurrently, genes involved in the flavonoid biosynthetic branch, including chalcone synthase (*CHS*) and chalcone isomerase like protein (*CHIL*), were upregulated, which may provide carbon skeletons and precursors for flavonoid biosynthesis [53,54,55]. Additionally, the expression levels of genes encoding flavonoid biosynthetic enzymes, including flavanone 3-hydroxylase/flavanonol synthase-related enzymes (*FHT*), flavonol synthase (*FLS*), and dihydroflavonol 4-reductase (*DFR*), were significantly increased. Based on these combined transcriptomic and metabolomic observations, we suggest that transcriptional activation of the flavonoid biosynthetic pathway may contribute to enhanced antioxidant capacity in lettuce under drought stress [56,57], although the accumulation of specific antioxidant flavonoids was not confirmed in the present untargeted metabolomics analysis.

Taken together, these transcriptomic and metabolomic data suggest a differential regulation of the phenylpropanoid pathway in FSO7-inoculated lettuce under drought: the lignin-associated branch was downregulated, whereas the flavonoid biosynthetic branch was activated. This apparent shift in the balance between these two branches may contribute to the transcriptional activation of the flavonoid biosynthetic branch and could be part of the mechanism associated with improved drought tolerance in FSO7-inoculated plants.

### 3.4. Limitations

The present study still has certain limitations. Although transcriptomic and metabolomic analyses have established a comprehensive molecular mechanism framework for FSO7-mediated drought tolerance in lettuce, key physiological indicators including soluble sugar, free proline, reactive oxygen species, malondialdehyde, antioxidant enzyme activities and photosynthetic physiological parameters were not determined. Meanwhile, the colonization dynamics of FSO7 in the rhizosphere and root tissues of lettuce were not quantified. These physiological indices and bacterial colonization characteristics are critical links to connect omics-level molecular changes with physiological drought tolerance phenotypes, and to clarify the intrinsic relationship between bacterial colonization and host stress responses in future studies. In addition, a full integrated analysis of the well-watered FSO7 treatment was not performed, as the present study focused on drought alleviation; such an analysis would help distinguish drought-specific from general PGPR-induced metabolic reprogramming and will be pursued in future work. Furthermore, because the present study was conducted under pot conditions, the applicability of our findings to field conditions remains to be further validated.

## 4. Materials and Methods

### 4.1. Plants, Strain Materials, and Growth Conditions

*Pseudomonas fluorescens* FSO7, *Pseudomonas fluorescens* FSO11 and *Bacillus brevis* CR30 were maintained in the Laboratory of Resource and Environmental Microbiology, College of Biological Science and Technology, Beijing Forestry University. The three strains were identified by 16S rRNA gene sequencing. Genomic DNA was extracted, and the 16S rRNA gene was amplified using universal primers 27F (5′-AGAGTTTGATCCTGGCTCAG-3′) and 1492R (5′-TACGGCTACCTTGTTACGACTT-3′). The purified PCR products were sequenced by Sanger dideoxy sequencing at Beijing Ruiboxingke Biotechnology Co., Ltd. (Beijing, China). The obtained sequences were compared with reference sequences in the NCBI GenBank database using BLASTN (version 2.17.0+; NCBI, Bethesda, MD, USA), and phylogenetic trees were constructed using the neighbor-joining method in MEGA (version 12.0) with 1000 bootstrap replicates (Figure S8). The 16S rRNA gene sequences of FSO7, FSO11, and CR30 have been deposited in GenBank under accession numbers QB034337, QB034338, and QB034339, respectively.

The lettuce cultivar used in this study was ‘American Fast’. The soil for the experimental planting was the sandy soil around the roots of A. ordosica in the Mu Us Sandy Land (37.744′ N; 107.249′ E) of Yanchi, Ningxia, China. After air drying, the soil was passed through a 2 mm sieve to remove fine roots and visible plant residues.

Experiments were conducted in a greenhouse at the Plant Science Activity Center of Beijing Forestry University. The greenhouse experiment lasted 62 days from sowing. The growth temperature was maintained at 22–26 °C, and a 16 h light/8 h dark photoperiod was provided using multispectral plant-growth lamps. Lettuce seeds were initially sown in plug trays and then transplanted into 1-gallon plastic pots with an upper diameter of 15.5 cm, a lower diameter of 12 cm, and a height of 14 cm.

The PGPR traits screened in this study (phosphate solubilization, siderophore production, IAA production, and ACC deaminase activity, together with PEG-6000-based drought tolerance assessment) were assayed following previously described protocols [58,59]. Detailed results are provided in Table S1. These analyses were performed as preliminary selection steps to justify the choice of candidate strains for the pot experiment and were not part of the main experimental workflow.

### 4.2. Drought Treatment and Experimental Design

The soil water gradient in this pot experiment was established with reference to the local soil water content of the Mu Us Sandy Land. A soil water content of 9% represented sufficient water, accounting for approximately 60% of field water capacity; whereas 3% represented drought stress, corresponding to roughly 20% of field water capacity [60]. The total pot weight was recorded every two days, and water was replenished to maintain the target soil water content.

Strains FSO7, FSO11, and CR30 were individually inoculated into TSB liquid medium (Beijing Aoboxing Biotechnology Co., Ltd., Beijing, China) and cultured overnight at 30 °C with shaking to obtain pure bacterial suspensions. The cultures were centrifuged to collect cell pellets, which were then resuspended in sterile water and adjusted to an optical density at 600 nm (OD_600_) of 0.5, corresponding to an effective concentration of approximately 1 × 10^8^ CFU·mL^−1^. The suspensions were then diluted 100-fold with sterile water to obtain the final inoculation solution (approximately 1 × 10^6^ CFU·mL^−1^). Each pot received 50 mL of the diluted suspension, resulting in a final inoculation dose of approximately 5 × 10^7^ CFU per pot (approximately 3.3 × 10^4^ CFU·g^−1^ soil).

To fully characterize the functional roles of the strains, we established a combined microbial agent treatment in this study. Theoretically, the combination of strains from different genera can achieve complementary plant growth-promoting functions, thereby conferring superior growth-promoting effects and stress tolerance benefits to host plants compared to single strains [61,62]. Pairwise antagonism among FSO7, FSO11, and CR30 was assessed by the cross-streak method on TSB agar plates [63]. Briefly, each strain was streaked perpendicular to the other and incubated at 30 °C for 48 h. The ability to grow at the intersection was recorded as the criterion for antagonism. The results showed a clear antagonistic interaction between FSO7 and CR30, rendering them incompatible for combined formulation. In contrast, no antagonistic effect was observed between FSO11 and CR30. Given that both FSO7 and FSO11 belong to the same species *P. fluorescens*, co-application of these two strains would likely result in functional redundancy. Therefore, the CR30+FSO11 combination was selected for the construction of the composite microbial agent. Specifically, the suspensions of CR30 and FSO11 were separately adjusted to OD_600_ = 0.5 (effective concentration 1 × 10^8^ CFU·mL^−1^), mixed in equal volumes (1:1, *v*/*v*), and then diluted 100-fold with sterile water to obtain the final inoculation solution (approximately 1 × 10^6^ CFU·mL^−1^). Each pot received 50 mL of the diluted suspension, resulting in a final inoculation dose of approximately 5 × 10^7^ CFU per pot (approximately 3.3 × 10^4^ CFU·g^−1^ soil).

Lettuce seeds of uniform size and complete development were selected, surface-sterilized by immersion in 2% NaOH solution for 15 min, and subsequently rinsed five times thoroughly with deionized water to remove residual alkali. The sterilized seeds were wrapped in sterile gauze, soaked in distilled water for 2 h, placed in sealed sterile bags, and stratified at 4 °C for 2 d. They were then sown in seedling trays. Upon reaching the three-leaf stage, healthy and uniformly growing seedlings without visible damage were selected and transplanted into plastic pots, each containing 1500 g of sandy soil collected from the rhizosphere of *Artemisia ordosica* in the Mu Us Sandy Land. One seedling was transplanted per pot. One week after transplantation, each inoculated treatment was irrigated with 50 mL of the corresponding bacterial suspension, while the blank control group received an equal volume of sterile water to ensure uniform water input across all treatments. Two weeks after inoculation, drought stress was imposed by establishing a water gradient according to the soil moisture characteristics of the Mu Us Sandy Land.

A two-factor completely randomized design was adopted for this pot experiment. The treatments included a normal-watering non-inoculated control (N-N), a drought-stressed non-inoculated control (N-D), and single-strain inoculations with FSO7, FSO11, and CR30, as well as the combined CR30+FSO11 inoculation, under both adequate-water and drought-stress conditions. Each treatment comprised 15 biological replicates, and all pots were randomly rearranged weekly to minimize positional bias within the greenhouse.

### 4.3. Sample Collection and Growth Traits Measurement

After two weeks of drought stress treatment, seedlings from the bacterial treatment group exhibiting the optimal growth-promoting effect (among which the FSO7 treatment group performed most prominently) were selected for subsequent analyses. Lettuce leaves at comparable developmental stages were randomly collected from the FSO7-treated group, thoroughly rinsed with sterile water to remove soil and surface adherents, blotted dry with sterile filter paper, promptly transferred into sterile centrifuge tubes, and flash-frozen in liquid nitrogen. The samples were then sent to Shanghai Personal Biotechnology Co., Ltd. for transcriptomic and metabolomic sequencing.

Concurrently, to evaluate the plant growth-promoting phenotype of the strain, six intact plants were retained from both the FSO7 treatment group and the control group for growth parameter determination. The specific measurement procedures were as follows: whole-plant length (measured from the apex of the uppermost functional leaf to the tip of the main root), root length (measured from the base of the stem to the tip of the longest root) and stem diameter (measured at the basal node) were determined using an electronic vernier caliper with a precision of 0.01 mm; fresh weight was obtained by weighing the whole plant, after which the plants were dried in an oven at 70 °C to constant weight, and dry weight was measured using an electronic analytical balance with a precision of 0.0001 g. For the multi-strain comparison involving all five treatments, three biological replicates per treatment were used for growth trait measurements. For the direct comparison between the FSO7-inoculated and uninoculated groups, six biological replicates were retained to increase statistical power. All samples were collected from the same batch of plants.

### 4.4. cDNA Library Preparation and Transcriptome Analysis

Lettuce samples were taken in 2 mL grinding tubes and thoroughly ground in a grinder at 60 Hz for 60 s. Total RNA was extracted using TRIzol reagent according to the manufacturer’s instructions. RNA purity was assessed using NanoDrop 2000 detection (Thermo Scientific, Waltham, MA, USA), and RNA integrity was evaluated by agarose gel electrophoresis. Total RNA was used as input for RNA-seq library preparation with the NEBNext Ultra II RNA Library Prep Kit (New England Biolabs, Ipswich, MA, USA). According to the manufacturer’s instructions, mRNA was enriched by oligo(dT) beads, interrupted by divalent cation, and reverse transcribed to cDNA using oligo(dT) as a template and random oligonucleotide as a primer. The cDNA was purified, amplified by PCR, and purified again to obtain the final library. The sequencing library was then sequenced on the NovaSeq 6000 platform (Illumina, San Diego, CA, USA) by Shanghai Personal Biotechnology Co., Ltd (Shanghai, China). Raw fluorescence image files generated by sequencing were converted into FASTQ raw data using software integrated into the sequencing platform. Raw reads were filtered using fastp v0.22.0 to obtain clean reads.

A total of 76.14 GB of clean data was obtained from 12 lettuce leaf samples (*n* = 3). All Q20 and Q30 values exceeded 98.7% and 96.4%, respectively, indicating highly reliability of the sequencing results (Table S2). Clean reads from each sample were aligned to the lettuce reference genome GCF_002870075.4_Lsat_Salinas_v11: https://ftp.ncbi.nlm.nih.gov/genomes/all/GCF/002/870/075/GCF_002870075.4_Lsat_Salinas_v11/ (accessed on 15 January 2026). Gene expression levels were quantified using HTSeq (version 2.0.2) and normalized as FPKM (fragments per kilobase of transcript per million mapped reads). Differentially expressed genes (DEGs) were identified using DESeq2 (version 1.42.0) with thresholds of |log_2_(fold change) | ≥ 1 and unadjusted *p* < 0.05 for primary screening. The Benjamini–Hochberg adjusted *p*-values (padj) were also calculated; due to the limited sample size (*n* = 3), applying padj < 0.05 resulted in a substantial reduction in DEG, and the complete list of DEGs with their padj values is provided in Table S7. GO and KEGG enrichment analyses were performed using clusterProfiler (version 4.6.0), with a significance threshold of *p* < 0.05.

### 4.5. Quantitative Real-Time PCR

Quantitative real-time PCR (qRT-PCR) was used to validate the RNA-seq data. Nine differentially expressed genes (DEGs) were selected for qRT-PCR validation, spanning different pairwise comparisons, both up- and down-regulated directions, and a broad range of fold-change magnitudes. *ACT7* (LOC111882438) was used as the internal reference gene. Each qRT-PCR value was calculated from three biological replicates per treatment group, each with three technical replicates. Primers were designed using Primer 6.0, and primer sequences are listed in Table S5. Total RNA was reverse transcribed into cDNA by reverse transcription reagent (Advantage^®^ RT-for-PCR Kit, Takara, Beijing, China) as described above. qRT-PCR was performed using AceQ^®^ qPCR SYBR^®^ Green Master Mix Q111-02 (Vazyme, Nanjing, China), and the reaction system and cycling conditions were set according to the kit instructions. Relative gene expression levels were determined by the 2^−ΔΔCt^ method [64].

To assess the agreement between RNA-seq and qRT-PCR, the log_2_(fold change) values obtained from the two platforms were compared for the nine selected genes. Linear regression was performed with RNA-seq log_2_(FC) as the independent variable and qRT-PCR log_2_(FC) as the dependent variable, and the coefficient of determination (R^2^) was calculated to quantify the agreement between the two platforms. All analyses were performed in GraphPad Prism 9.0.0 (GraphPad Software, San Diego, CA, USA).

### 4.6. Untargeted Metabolomic Analysis

Forty milligrams of the ground lettuce sample was weighed in a 2 mL centrifuge tube, followed by the addition of 300 μL of the precooled extract solution (methanol: acetonitrile: water = 2:2:1, *v*/*v*/*v*). The mixture was vortexed and homogenized twice in a high-throughput tissue grinder at 55 Hz for 60 s. After homogenization, samples were sonicated in an ultrasonic cleaner for 10 min at 4 °C and incubated at −20 °C for 30 min to precipitate proteins. Samples were then centrifuged at 12,000 rpm for 10 min at 4 °C. The supernatant was collected, filtered through a 0.22-μm membrane, and transferred to injection vials for mass spectrometry. For quality control (QC), 10 μL of filtrate from each sample was pooled and thoroughly mixed to prepare a pooled QC sample.

Untargeted metabolomic analysis was performed using a liquid chromatography-mass spectrometry (LC-MS) system, with all experiments conducted by Shanghai Paisenuo Biotech Co., Ltd. Chromatographic separation was achieved on a Thermo Vanquish Flex (Thermo Scientific, Waltham, MA, USA) equipped with an ACQUITY UPLC HSC-T3 column (Waters, Milford, MA, USA). The mobile phase consisted of 0.1% formic acid in water (solvent A) and acetonitrile containing 0.1% formic acid (solvent B). Sample measurements were performed using a gradient program with a flow rate set at 0.4 mL min^−1^. The column temperature was set at 40 °C, the autosampler temperature was 8 °C, and the injection volume was 2 μL. The effluent was introduced into a Thermo Orbitrap Exploris 120 mass spectrometer (Thermo Scientific, Waltham, MA, USA) equipped with a heated electrospray ionization source for metabolite detection.

Raw metabolomic data were converted to .mzXML format using ProteoWizard (version 3.0.24000) and processed using XCMS v3.12.0 for peak alignment, retention time correction, and peak area extraction. Metabolites were annotated using the PSNGM (PerSonalbio Next-Generation Metabolomics Database) in combination with public databases (HMDB, Metlin, MassBank, mzCloud). The annotation criteria were: mass error < 10 ppm, MS/MS spectral matching (>70), and a collision energy of 30% (HCD). The annotation confidence levels were assigned according to the Metabolomics Standards Initiative (MSI) guidelines, and the majority of reported metabolites were annotated at Level 2. For quality control, features with RSD > 30% in QC samples or missing values > 50% in any group were removed. Blank subtraction was not performed. Metabolite abundances were normalized and log_2_-transformed. Differentially accumulated metabolites (DAMs) were screened using |log_2_(fold change)| > 0.5, VIP > 1 from the OPLS-DA model, and unadjusted *p* < 0.05. The Benjamini–Hochberg adjusted *p*-values (padj) were also calculated, and the number of DAMs identified using padj < 0.05 changed only slightly compared to the unadjusted analysis, indicating that the metabolomic data are highly robust. The complete list of DAMs with their padj values is provided in Table S8, and the complete annotation table of metabolites is provided in Table S9.

### 4.7. Integration of Transcriptomic and Metabolomic Data

Metabolite abundances were normalized and log_2_ transformed, while gene counts were normalized using the DESeq2 method. Then based on these two types of normalized data, Pearson correlation coefficients (PCC) between differentially accumulated metabolites (DAMs) and differentially expressed genes (DEGs) then calculated in R (version 4.3.2; http://www.r-project.org/) (accessed on 10 January 2026). A nine-quadrant plot was generated to visualize the correlation patterns between DEGs and DAMs based on their log_2_ fold changes. Gene-metabolite pairs with PCC ≥ 0.8 were retained for subsequent KEGG pathway enrichment analysis, which was performed using the clusterProfiler package in R with lettuce (*Lactuca sativa*) as the reference species and a threshold of *p* < 0.05.

### 4.8. Statistical Analysis

GraphPad Prism 9.0.0 was used for statistical analyses of growth and physiological indicators. For growth traits, a two-way analysis of variance (ANOVA) was performed with water condition (W) and inoculation treatment (I) as fixed factors, including their interaction (W × I). When a significant interaction or main effect was detected, Tukey’s multiple-comparison test was used for post hoc comparisons among the multiple inoculation treatments, while Sidak’s multiple-comparison test was used for pairwise comparisons between the FSO7-inoculated group and the uninoculated control within each water condition. *p* < 0.05 was considered statistically significant. Figures were generated using GraphPad Prism 9.0.0.

## 5. Conclusions

**Figure 7:**
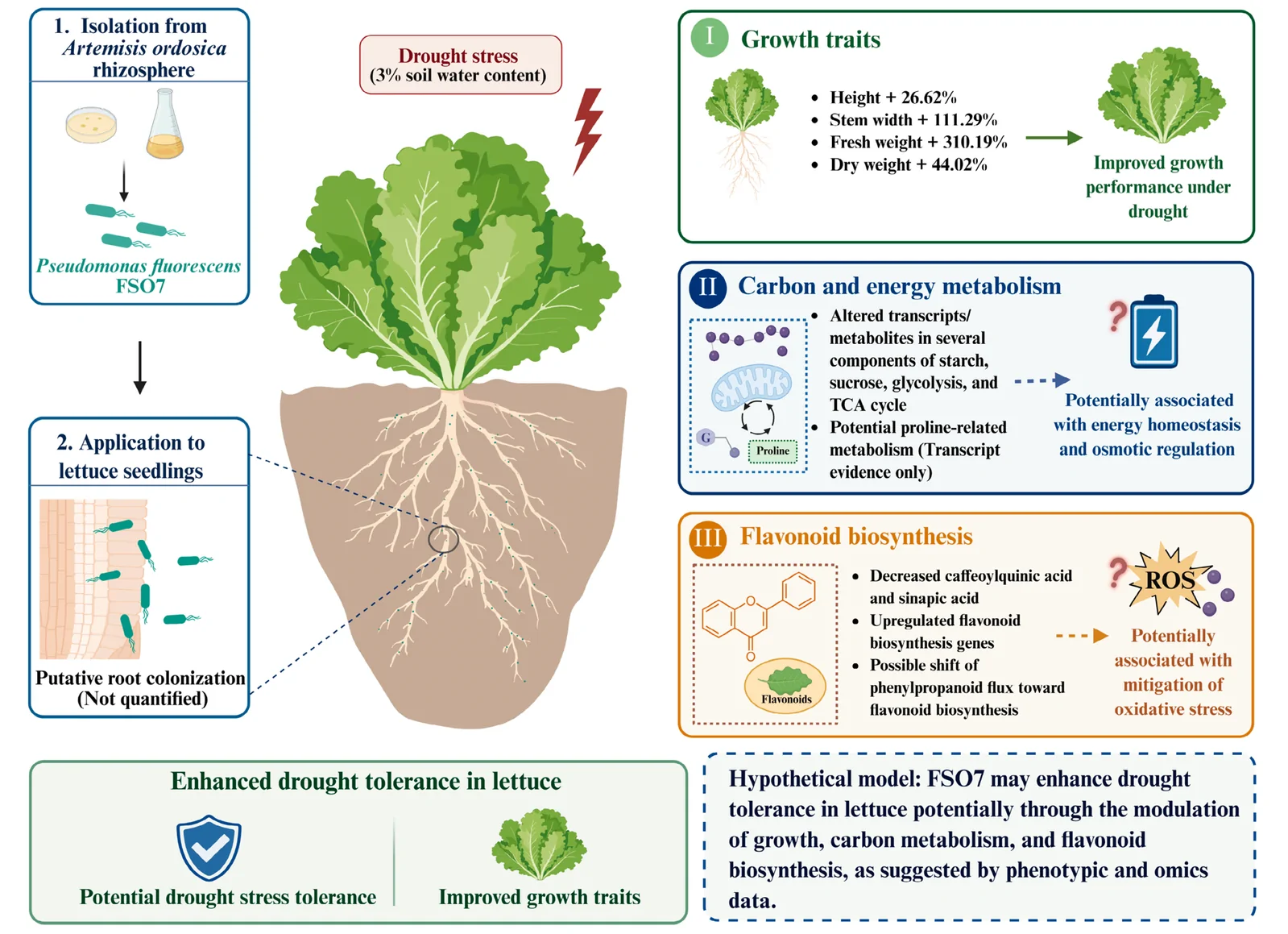
Hypothetical model summarizing the potential mechanisms by which FSO7 may enhance drought tolerance in lettuce. The model is based on phenotypic and omics data from this study. The left panel illustrates the experimental workflow, including the isolation of FSO7 from *Artemisia ordosica*, its application to lettuce seedlings, and subsequent drought stress treatment (3% soil water content). The right panel summarizes the potential mechanisms: under drought stress, FSO7-inoculated plants showed improved growth traits; concurrently, transcripts and metabolites related to carbon and energy metabolism were altered, and the flavonoid biosynthesis pathway was transcriptionally activated. These processes may collectively contribute to improved lettuce growth and potentially enhanced stress resilience. Solid arrows represent experimentally measured results, including improved growth traits and omics changes; dashed arrows indicate proposed associations or physiological effects; black arrows indicate the sequence of the experimental procedure; question marks (?) highlight key hypothetical steps requiring further validation. This schematic is intended to be hypothesis-generating, and all inferred relationships require further experimental validation. (Created in: https://BioRender.com) (accessed on 30 January 2026).

In this study, we systematically investigated the regulatory effects and molecular mechanisms of *Pseudomonas fluorescens* FSO7, isolated from the rhizosphere of the desert plant *Artemisia ordosica*, on lettuce drought adaptation by integrating pot experiments with transcriptomic and metabolomic analyses. FSO7 showed the strongest growth-promoting effect among the tested bacterial treatments under drought stress. Integrated metabolomic and transcriptomic analyses further revealed that FSO7 inoculation was closely associated with altered transcripts and metabolites related to carbon and flavonoid metabolism in lettuce. Specifically, pathways involved in starch and sucrose metabolism, glycolysis, and the TCA cycle were markedly changed, and the flavonoid biosynthetic pathway was activated at the transcriptional level (Figure 7). Based on these correlative omics evidence, we tentatively infer that FSO7 may contribute to improved lettuce growth under drought stress, in part, through the coordinated modulation of these metabolic processes.

Overall, this study provides a mechanistic framework for understanding the interaction between FSO7 and lettuce under drought stress. It also highlights the potential of desert plant-associated microorganisms as resources for sustainable agricultural development and provides a foundation for discovering, developing, and applying drought-tolerant microbial resources from desert plant rhizospheres.

## Figures and Tables

**Table 1:** Two-way ANOVA of growth traits in lettuce under different water and strain treatments (F values).

Source	df	Height	Root Length	Whole-Plant Fresh Weight	Shoot Fresh Weight
Water (W)	1	138.3 ****	0.1742	979.5 ****	455.0 ****
Strain (S)	4	9.056 ***	2.437	34.15 ****	9.490 ***
W × S	4	2.345	0.6762	21.89 ****	3.783 *

**Note:** F values are shown. * *p* < 0.05, *** *p* < 0.001, **** *p* < 0.0001. Water (W), water treatment (well-watered vs. drought); Strain (S), strain treatment (N(uninoculated), Pf, CR30, FSO11, CR30+FSO11); W × S, interaction between water and strain treatments. Each treatment combination consisted of three independent biological replicates (*n* = 3).

## Data Availability

Data are contained within the article and Supplementary Materials. The 16S rRNA gene sequences of strains FSO7, FSO11, and CR30 have been deposited in the NCBI GenBank database under accession numbers QB034337, QB034338, and QB034339. The raw transcriptome data were deposited in the National Center for Biotechnology Information (NCBI) with the accession number PRJNA1469410.

## Supplementary Materials

The following supporting information can be downloaded at: https://www.mdpi.com/article/10.3390/plants15193039/s1, Figure S1: Effects of FSO7 inoculation on lettuce growth under well-watered and drought stress conditions; Figure S2. Transcriptome profiles of lettuce under FSO7 inoculation and drought stress. (A) Correlation heat map of different samples of maize transcriptome sequencing; Figure S3: GO enrichment analysis of DEGs in representative comparisons; Figure S4: KEGG pathway enrichment of DEGs in the four pairwise comparisons; Figure S5: Validation of RNA-seq data by qRT-PCR; Figure S6: KEGG pathway enrichment of DAMs in the four pairwise comparisons; Figure S7: Integrated transcriptomic and metabolomic analysis of lettuce in the Pf-D vs. N-D comparison; Figure S8: Phylogenetic trees of the isolated strains based on 16S rRNA gene sequences. Table S1: Plant growth-promoting characteristics and drought tolerance of the tested strains; Table S2: Two-way ANOVA of growth traits in lettuce under water treatment and FSO7 inoculation (F values); Table S3: RNA-seq quality metrics and read-mapping statistics for lettuce leaf samples; Table S4: DEGs and DAMs associated with starch and sucrose metabolism, glycolysis, the TCA cycle, and proline-related metabolism in the Pf-D vs. N-D comparison; Table S5: DEGs and DAMs associated with phenylpropanoid and flavonoid biosynthesis in the Pf-D vs. N-D comparison; Table S6: Primer sequences used for qRT-PCR validation of differentially expressed genes; Table S7: Complete list of differentially expressed genes (DEGs) in the Pf-D vs. N-D comparison; Table S8: Complete list of differentially accumulated metabolites (DAMs) in the Pf-D vs. N-D comparison; Table S9: Complete annotation table of metabolites in lettuce leaves-POS/NEG.
