# Supplementary material for: Enhancement of Drought Stress Tolerance in Lettuce by Pseudomonas fluorescens FSO7 Isolated from Artemisia ordosica Rhizosphere
Source: Plants (Basel). 2026 Oct 4;15(19):3039. doi: 10.3390/plants15193039 (PMC13666857; doi:10.3390/plants15193039)
Supplement: Supplementary file 1 [file plants-15-03039-s001.zip › Supplementary material-Figure.pdf]

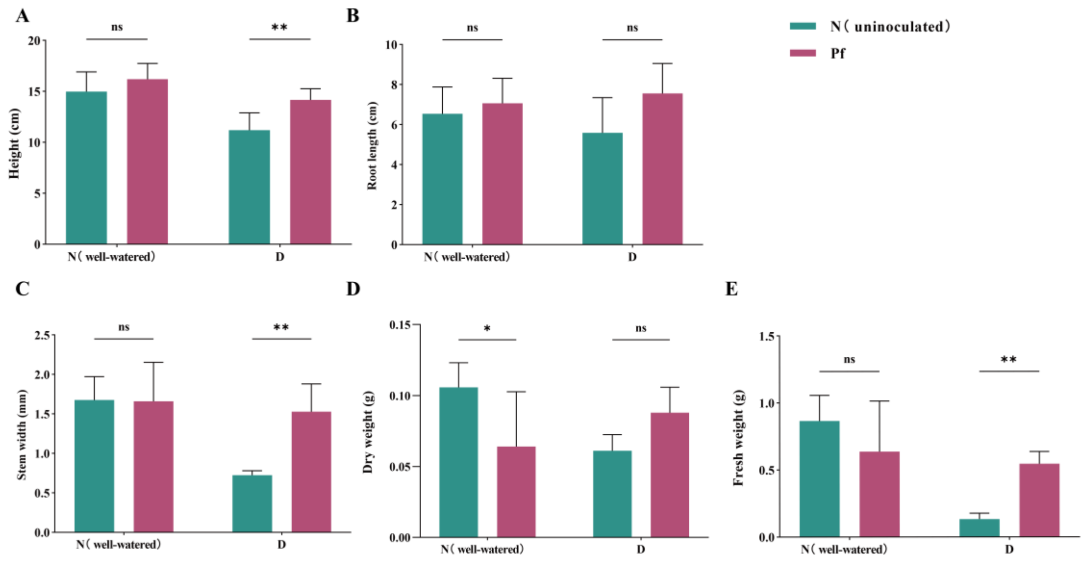

Figure S1. Effects of FSO7 inoculation on lettuce growth under well-watered and drought stress conditions. (A) Plant height, (B) root length, (C) stem width, (D) dry weight, and (E) fresh weight of lettuce under uninoculated well-watered (N well-watered), FSO7-inoculated well-watered (Pf well-watered), uninoculated drought (N D), and FSO7-inoculated drought (Pf D) treatments. Error bars indicate SD (n = 6 biological replicates). Two-way ANOVA was used to assess the effects of water condition (W), inoculation treatment (I), and their interaction (W × I). Sidak's multiple-comparison test was used for pairwise comparisons between the FSO7-inoculated group and the uninoculated control within each water condition. Asterisks indicate significant differences between N and Pf treatments (ns, not significant; *$P < 0.05$; **$P < 0.01$; ***$P < 0.001$; ****$P < 0.0001$).

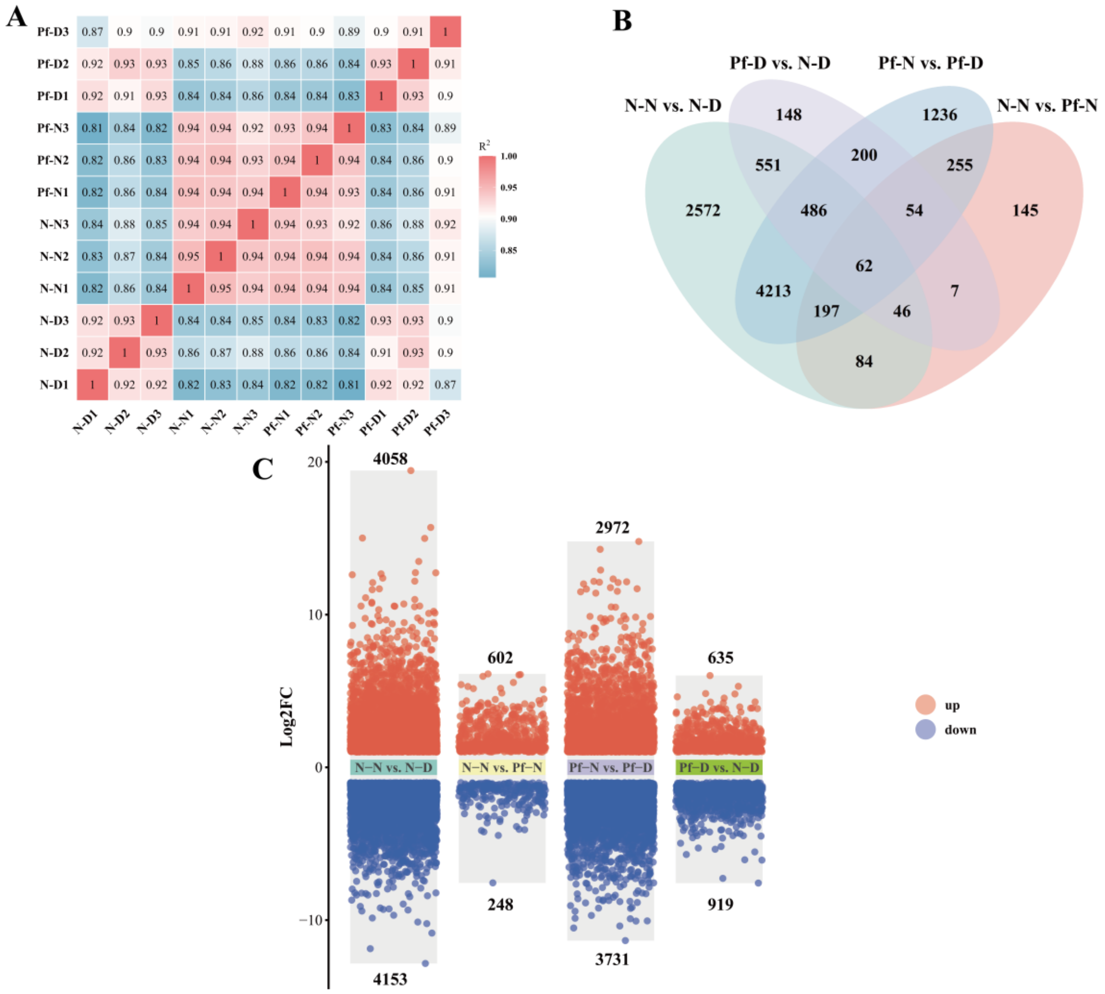

Figure S2. Transcriptome profiles of lettuce under FSO7 inoculation and drought stress. (A) Correlation heat map of different samples of maize transcriptome sequencing. (B) Venn diagram showing the overlap of DEGs among pairwise comparisons. (C) DEGs in the N-N vs. N-D, N-N vs. Pf-N, Pf-N vs. Pf-D, and Pf-D vs. N-D comparisons. Numbers above and below each comparison indicate upregulated and downregulated genes.

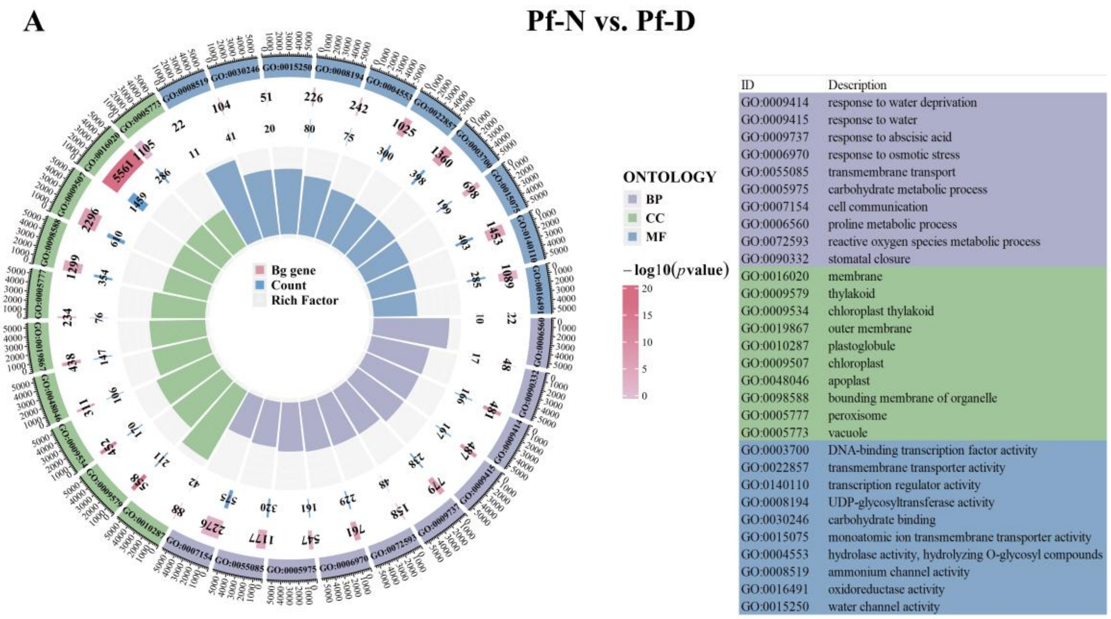

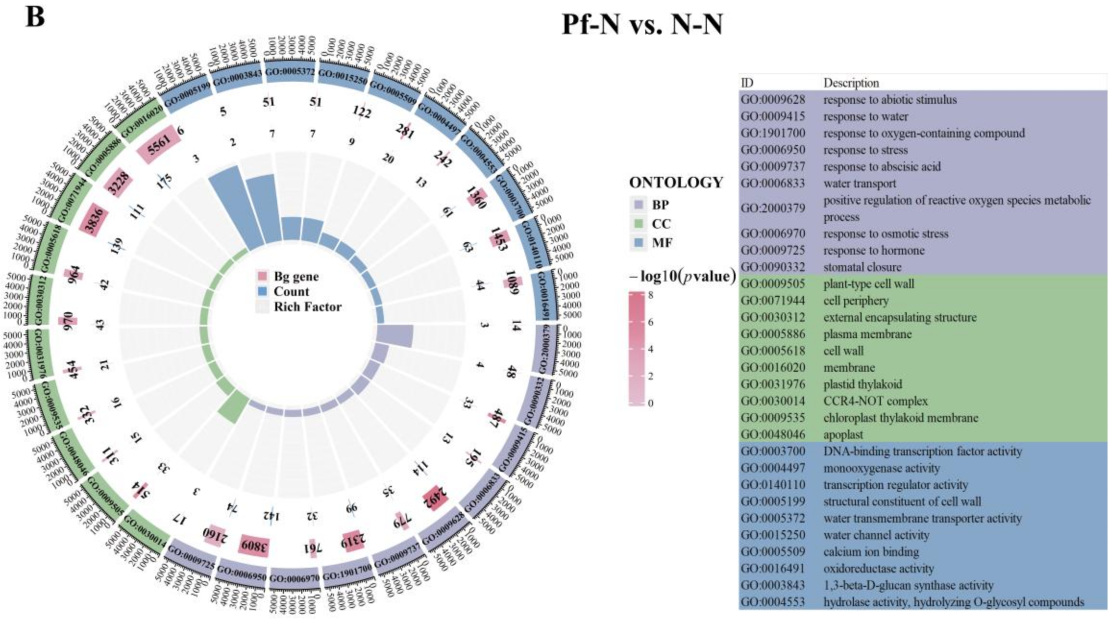

Figure S3. GO enrichment analysis of DEGs in representative comparisons. (A) Pf-N vs. Pf-D. (B) Pf-N vs. N-N. BP, biological process; CC, cellular component; MF, molecular function.

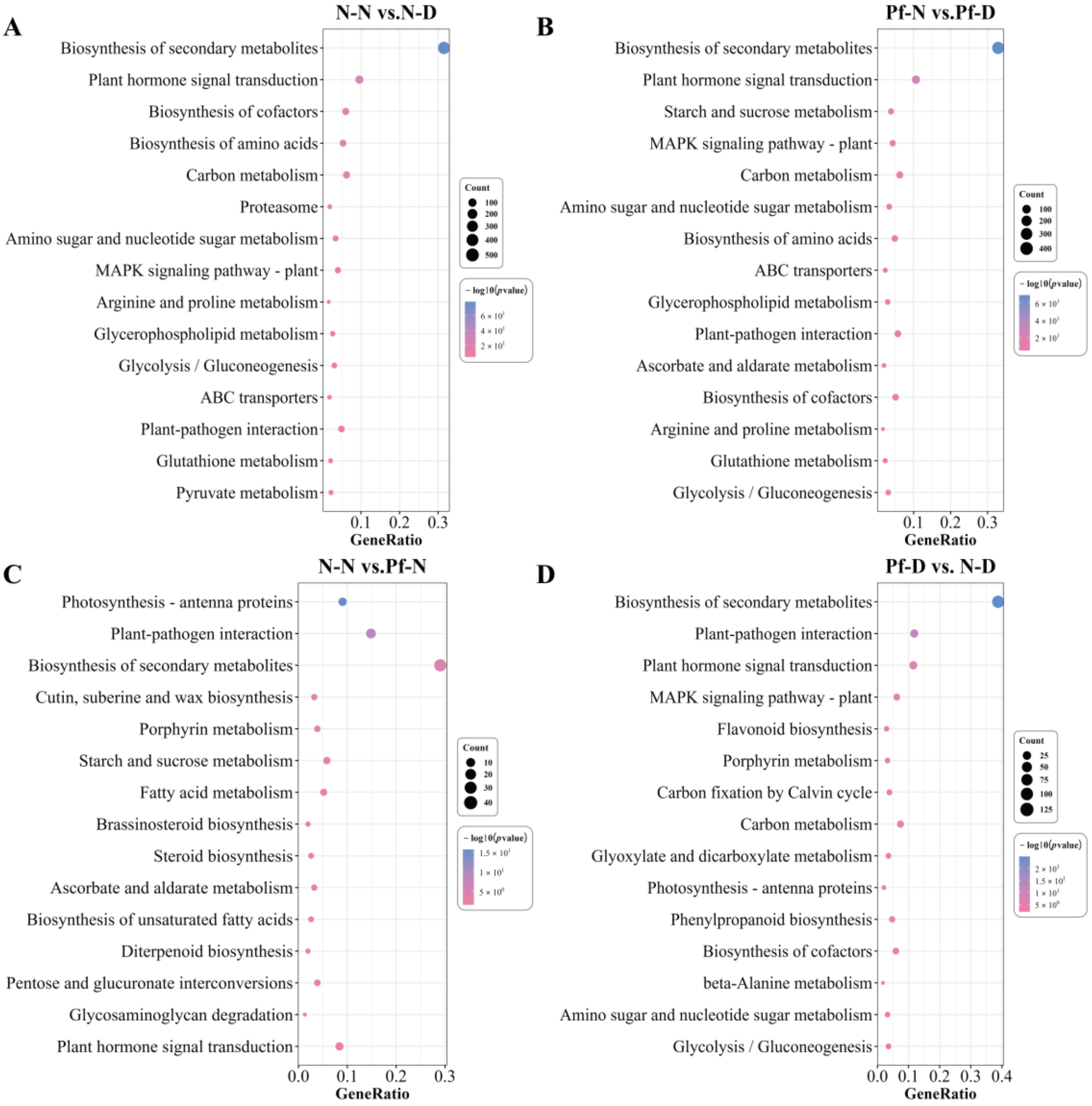

Figure S4. KEGG pathway enrichment of DEGs in the four pairwise comparisons. (A) N-N vs. N-D. (B) Pf-N vs. Pf-D. (C) N-N vs. Pf-N. (D) Pf-D vs. N-D. Bubble size indicates the number of DEGs enriched in each pathway, and color indicates enrichment significance.

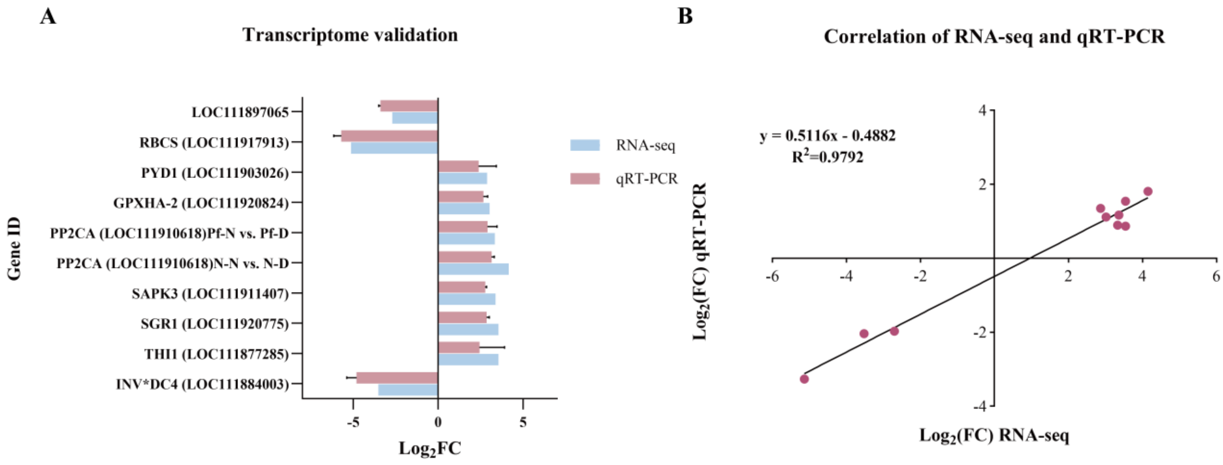

Figure S5. Validation of RNA-seq data by qRT-PCR. (A) Comparison of $\log_2$(FC) values obtained by RNA-seq and qRT-PCR for nine selected DEGs. RNA-seq values are single estimates derived from three biological replicates; qRT-PCR values are shown as mean ± SD (*n* = 3 biological replicates per group, each with three technical replicates). Error bars are shown for the qRT-PCR data only. (B) Linear regression of RNA-seq $\log_2$(FC) against qRT-PCR $\log_2$(FC) for the nine genes ($y = 0.5116x - 0.4882$, $R^2 = 0.9792$).

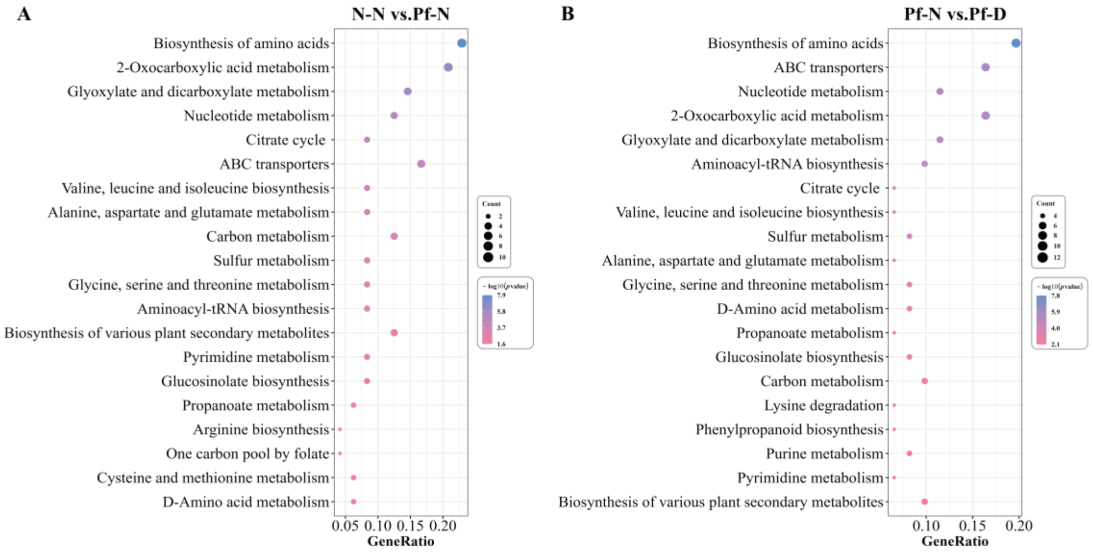

Figure S6. KEGG pathway enrichment of DAMs in the four pairwise comparisons. (A) N-N vs. Pf-N. (B) Pf-N vs. Pf-D. Bubble size indicates the number of DAMs enriched in each pathway, and color indicates enrichment significance.

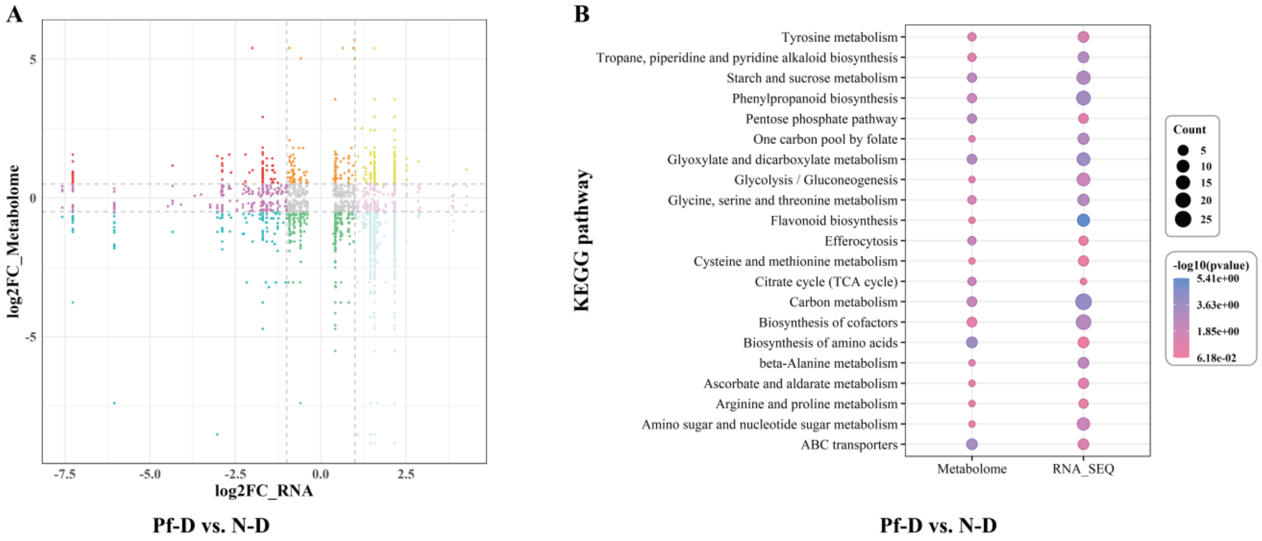

Figure S7. Integrated transcriptomic and metabolomic analysis of lettuce in the Pf-D vs. N-D comparison. (A) Nine-quadrant plot showing correlations between DEGs and DAMs. (B) KEGG enrichment bubble plot of co-enriched transcriptomic and metabolomic pathways in the Pf-D vs. N-D comparison.

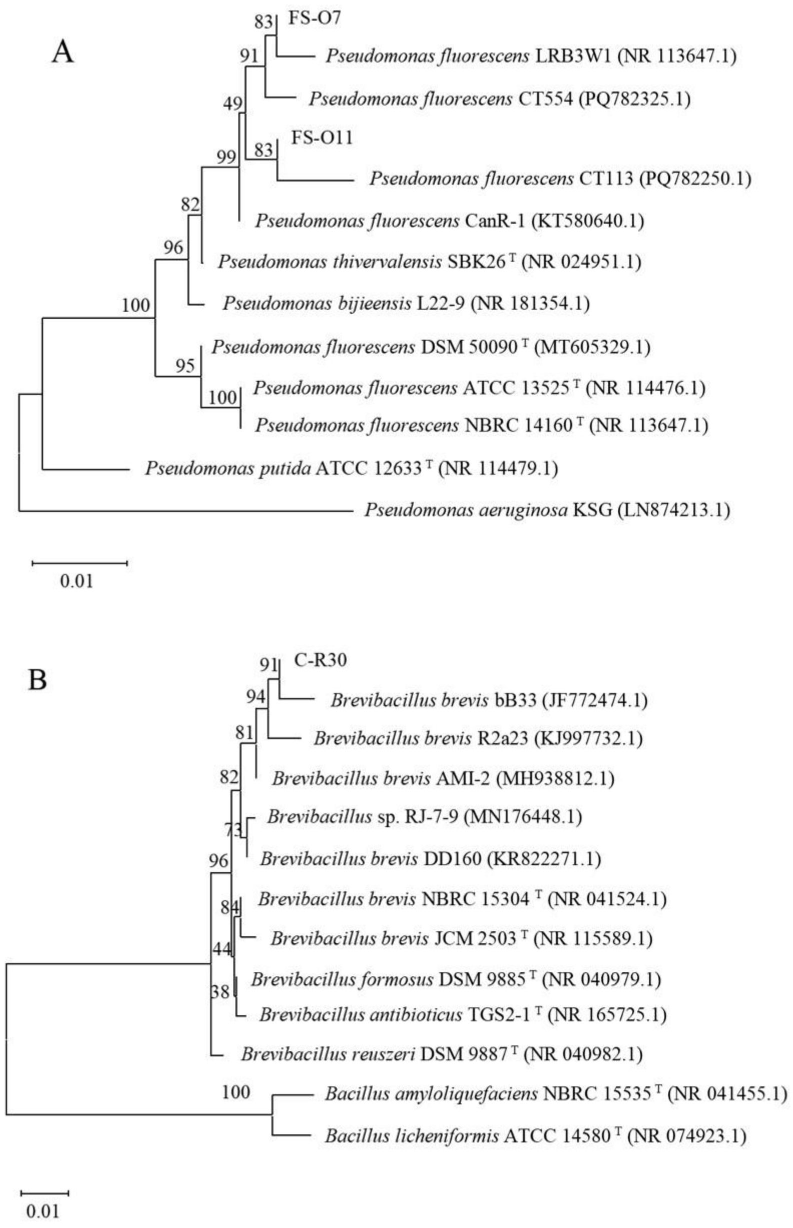

Figure S8. Phylogenetic trees of the isolated strains based on 16S rRNA gene sequences. (A) Phylogenetic tree showing the relationship between strain FSO7, FSO11, and representative *Pseudomonas* species. (B) Phylogenetic tree showing the relationship between strain CR30 and representative *Brevibacillus* species. The trees were constructed using the neighbor-joining method in MEGA 12.0. The 16S rRNA gene sequences of the reference strains were retrieved from the NCBI GenBank database, and their accession numbers are shown in parentheses. *Pseudomonas aeruginosa* KSG and *Bacillus licheniformis* ATCC 14580 were used as outgroups for (A) and (B), respectively. The scale bar represents 0.01 substitutions per nucleotide position.
