# Supplementary material for: Enhancement of Drought Stress Tolerance in Lettuce by Pseudomonas fluorescens FSO7 Isolated from Artemisia ordosica Rhizosphere
Source: Plants (Basel). 2026 Oct 4;15(19):3039. doi: 10.3390/plants15193039 (PMC13666857; doi:10.3390/plants15193039)
Supplement: Supplementary file 1 [file plants-15-03039-s001.zip › Supplementary material-Table.pdf]

Table S1 Plant growth-promoting characteristics and drought tolerance of the tested strains

	**FSO7**	**FSO11**	**CR30**
**Nitrogen fixation**	+	+	+
**Siderophore production (D/d)**	2.97±0.28	2.63±0.16	2.15±0.13
**Organic phosphate solubilization**	+	+	+
**IAA production (mg/L)**	7.38±1.24	21.82±1.9	28.05±2.1
**Potassium solubilization**	-	-	-
**ACC deaminase production**	+++	+++	++
**Inorganic phosphate solubilization**	-	++	-
**OD_{600}（20% PEG）**	0.42±0.008	0.42±0.014	0.2±0.005
**OD_{600}（40% PEG）**	0.12±0.01	0.11±0.004	0.3±0.018
**OD_{600}（60% PEG）**	0.02±0.001	0.12±0.007	0.16±0.012

Note: "+", weak; "++", moderate; "+++", strong; "-", not detected. D/d, ratio of halo diameter to colony diameter (semi-quantitative).

Table S2 Two-way ANOVA of growth traits in lettuce under water treatment and FSO7 inoculation (F values)

Source	df	Height	Root length	Stem width	Dry weight	Fresh weight
Water(W)	1	19.67***	0.1468	15.26***	1.148	21.42***
Strain(S)	1	10.27**	4.276	8.104**	0.5821	1.066
W×S	1	1.792	1.792	8.804**	12.54**	12.95**

Note: F values are shown. *p < 0.05, **p < 0.01, ***p < 0.001. Water (W), water treatment (well-watered vs. drought); Strain (S), strain treatment (uninoculated control vs. FSO7 inoculation); W × S, interaction between water and strain treatments. Each treatment combination consisted of six independent biological replicates (*n* = 6).

Table S3 RNA-seq quality metrics and read-mapping statistics for lettuce leaf samples.

Sample	Raw Reads No	Clean Reads No	Q20(%)	Q30(%)	Total_Mapped	GB
Pf-N1	44478872	43882136	98.92	96.8	42899497 (97.76%)	6.153685925
Pf-N2	44244948	43655756	98.88	96.71	42657787 (97.71%)	6.125058789
Pf-N3	47177526	46492884	98.88	96.73	45511250 (97.89%)	6.525136019
Pf-D1	40711672	40088876	98.79	96.48	39261032 (97.93%)	5.62126036
Pf-D2	46123400	45433470	98.82	96.59	44433055 (97.80%)	6.372047964
Pf-D3	46713030	46006180	98.85	96.67	44810968 (97.40%)	6.456380336
N-D1	54852428	54144884	99.08	97.31	52964268 (97.82%)	7.596957578
N-D2	51342622	50580264	98.85	96.61	49563097 (97.99%)	7.102568152
N-D3	38439000	37825840	98.77	96.43	37012907 (97.85%)	5.307023612
N-N1	43827502	43140204	98.8	96.5	42136562 (97.67%)	6.052322298
N-N2	46653228	46048568	98.92	96.83	44916618 (97.54%)	6.461850073
N-N3	46013406	45379916	98.87	96.69	44275777 (97.57%)	6.366470274

Table S4 DEGs and DAMs associated with starch and sucrose metabolism, glycolysis, the TCA cycle, and proline-related metabolism in the Pf-D vs. N-D comparison.

Gene/metabolite	ID	log2FoldChange	regulated
FBA2	LOC111913347	3.18941584	up
*INV*DC4*	LOC111883991	2.822218787	up
D-Glucose	MN2925(NEG)	-1.291780015	down
TPPD	LOC111899324	-2.414733653	down
LOC111886178	-	2.354824881	up
SS3	LOC111896254	1.50009612	up
WAXY	LOC111911063	2.433404966	up
ISA2	LOC111886399	1.162162988	up
BMY1	LOC111890627	3.495448533	up
Citrate	MN3214	0.854271408	up
Isocitrate	MN1422	0.815655525	up
P4H10	LOC111915462	-1.255022042	down

Table S5 DEGs and DAMs associated with phenylpropanoid and flavonoid biosynthesis in the Pf-D vs. N-D comparison.

Gene/metabolite	ID	log2FoldChange	regulated
PAL1	LOC111916376	-1.769289803	down
CYP84A1	LOC111895133	-2.795475311	down
Sinapic acid	MP7123(POS)	-0.688096014	down
	LOC111883451	3.24293106	up
CHS	LOC111882072	2.24374903	up
	LOC111883451	3.24293106	up
CHIL2	LOC111919658	4.272265264	up
CYP73A1	LOC111876528	-1.176372544	down
FHT	LOC111897600	1.860346088	up
DFR	LOC111897350	2.132096574	up
HST	LOC111914857	-2.829463947	down
FLS	LOC111900153	1.349242444	up
CYP75B2	LOC111891078	3.835529541	up
Caffeoyl quinic acid	MN7420(NEG)	-0.68735108	down

Table S6 Primer sequences used for qRT-PCR validation of differentially expressed genes.

Genes	Primer	Forward/Reverse primer (5'-3')
ACT7(LOC111882438)	F	ATCCACGAGACGACTTAT
	R	CATCCTATCAGCAATTCCA
*INV*DC4 (LOC111884003)*	F	ATGATTGGTATGCGATTG
	R	CCTTCTCTTCTTATGTTGAT
THI1 (LOC111877285)	F	AGCCGAGGACTTAATCAT
	R	GTGGTTCATTGCGACTAA
SGR1 (LOC111920775)	F	ATACAGAGATGAAGTGGTA
	R	TTGGTGAAGATGAAGAAC
SAPK3 (LOC111911407)	F	AGAGATAAGAAGACTAAGGA
	R	CAGATGTGTTGGAGTTAA
PP2CA (LOC111910618)	F	AGTAGTGAGTTGCGAGAA
	R	CGGTTATATCCTTGTCCATT
GPXHA-2 (LOC111920824)	F	ATTATCCTGAGTTGACAA
	R	TTCACATTAACCTTGCTA
PYD1 (LOC111903026)	F	GGTGTGATGATGTATGGAT
	R	TATGGTTGCGAAGTTATGA
RBCS (LOC111917913)	F	ATACTATGATGGACGCTACTG
	R	ACTCTCCGATCTCCTTCA
LOC111897065	F	GGAACTGGATTATCTTGATG
	R	AATCTTGGATGACACCTT
